# Reactivity umpolung of the cycloheptatriene core in hexa(methoxycarbonyl)cycloheptatriene

**DOI:** 10.3762/bjoc.22.2

**Published:** 2026-01-05

**Authors:** Dmitry N Platonov, Alexander Yu Belyy, Rinat F Salikov, Kirill S Erokhin, Yury V Tomilov

**Affiliations:** 1 N. D. Zelinsky Institute of Organic Chemistry, Russian Academy of Sciences, 47 Leninsky prosp., 119991 Moscow, Russian Federation; 2 Higher School of Economics National Research University, 101000 Moscow, Russian Federation

**Keywords:** azo shift, cascade reactions, cycloheptatriene, relief of antiaromaticity, umpolung

## Abstract

A reactivity umpolung approach for the derivatization of cycloheptatrienes was extended to hexa(methoxycarbonyl)cycloheptatriene, which forms the corresponding anion reactive towards electrophiles. Despite the presence of four potential reactive sites, the reactions mainly involve the initial electrophilic attack onto the α-position relative to the hydrogen atom. The selectivity is either due to the high stability of the α-nucleophilic conformer or due to the promotion by the adjacent ester group. Cascade reactions upon the target connection, if installed, include the formation of norcaradienes, dihydroindazoles, a tetracyclodecene and a hydrazonocycloheptatriene derivative.

## Introduction

**Figure 1:**
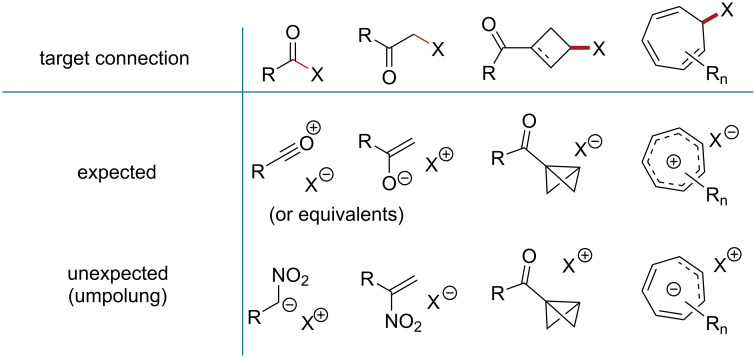
The expected and the unexpected in selected synthetic strategies.

Reactivity umpolung [1] is a synthetic concept in organic chemistry that contraposes the expected and the unexpected in terms of polarity in either reactions or synthetic strategies (Figure 1). Most effective reactions within this concept involve the in situ generation of a species exhibiting reversed polarity and the original group is reestablished at later reaction stages [2]. Other methods necessitate the umpolung modification and the reestablishment of the original group in separate reaction steps [3–4]. Additionally, there are approaches that represent only conceptual umpolung, utilizing synthetic equivalents that are not directly related to the target functional group; these equivalents can be transformed after the desired connection has taken place. For instance, nitroalkanes [1] and nitroalkenes [5–6] are regarded as equivalents of unavailable acyl anions and vinyloxenium cations, respectively. Reversed reactivity patterns also emerge due to non-uniform structures providing additional reaction pathways such as in bicyclobutanes (Figure 1) [7]. An umpolung approach was previously used in α-substitution of tropone [8].

Cycloheptatrienes are commonly expected to generate aromatic tropylium ions. Tropylium ions are so stable that they are formed not only through leaving-group elimination but through intermolecular hydride-transfer reactions as well. Therefore, the expected strategy to establish a chemical bond with a cycloheptatriene core is a reaction of a tropylium ion derivative with a nucleophile. Such reactions may involve either carbon [9–11] or heteroatom [12–16] nucleophiles (Figure 1). Reactions of cycloheptatrienyl anions with electrophiles present an unexpected approach as the anions possess antiaromatic nature and, therefore, appear to be unstable.

**Figure 2:**
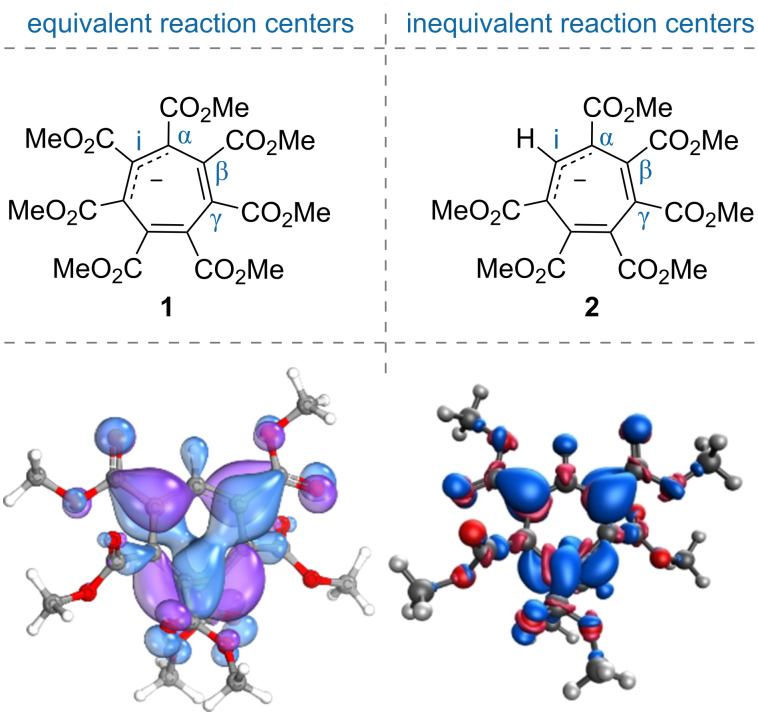
Distortion in antiaromatic hepta- and hexa(methoxycarbonyl)cycloheptatrienyl anions **1** and **2**. HOMO (bottom left) and Fukui *f*^–^ function (bottom right) shapes for anion **2**. The data were obtained at the r^2^SCAN-3c/CPCM(acetonitrile) level of theory.

Previously we demonstrated that stable hepta(methoxycarbonyl)cycloheptatrienyl potassium **1** (Figure 2) undergoes reactions with various electrophilic reagents which presented the first examples of unexpected pathways for the derivatization of cycloheptatrienes [17–19]. However, the anion is highly symmetric and does not challenge regioselectivity. Therefore, besides the unavailability of other stable cycloheptatrienyl anions until recently, its derivatives were not expected to show high selectivity in reactions. Herein, we present the first examples of selective reactions of low-symmetry hexa(methoxycarbonyl)cycloheptatrienyl anion **2** [20] towards electrophiles. Additionally, the antiaromatic properties of anion **2** [21] allow to regard these reactions as one of those driven by the relief of antiaromaticity [22–27].

## Results and Discussion

In our previous work [21], we demonstrated that most cycloheptatrienyl anions prefer conformers distorted to an allylic anionic system (shown as tautomer in Figure 2) with a diene fragment (shown as double bonds) twisted over it. In hexa(methoxycarbonyl)cycloheptatrienyl anion **2** the prevalent allylic conformer is the one with the hydrogen atom in the middle of the allyl-anionic fragment (the *i*-position) to afford the planarity of the two neighboring ester groups and thereby stabilize the anion. The prevalence of this isomer is also confirmed by the NMR data [21], however, other conformers with different charge distributions are also available in solution. To understand the nucleophilic reactivity of the most stable isomer of anion **2** we analyzed the structure of its highest occupied molecular orbital (HOMO) and the distribution of the electrophilic Fukui function (*f*^–^) [28] – the difference in electron density between the anion and the derived radical by abstraction of one electron (Figure 2). Both approaches revealed that the α-position is most nucleophilic. Notably, anion **1** demonstrated similar HOMO and Fukui function distribution (see Supporting Information File 1), however, all positions would lead to the very same products.

**Scheme 1:**
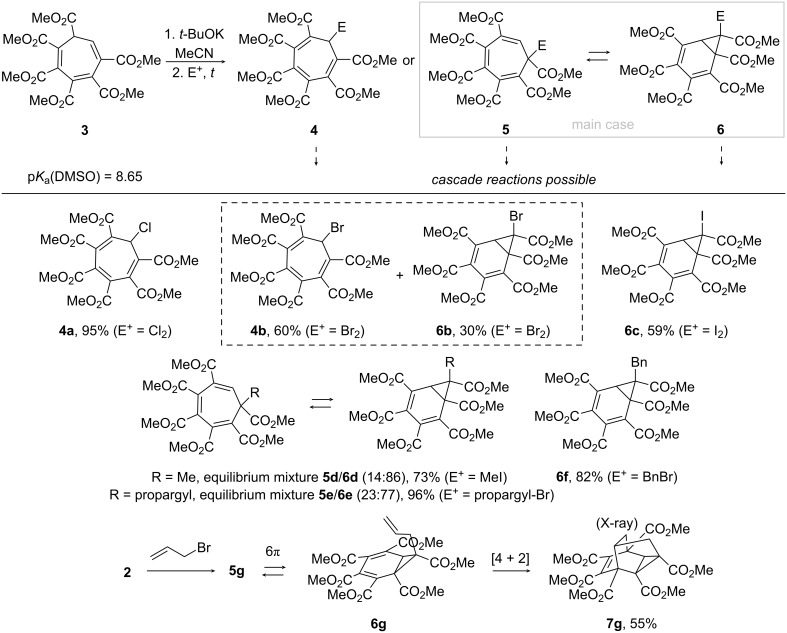
Reactions of anion **2** generated from cycloheptatriene **3** with halogens and alkyl halides.

The potassium salt of anion **2** was generated in an acetonitrile solution from hexa(methoxycarbonyl)cycloheptatriene **3** (p*K*_a_(DMSO) = 8.65) [20–21] with potassium *tert*-butoxide and subjected to reactions with electrophilic reagents. The reactions gave two primary products **4** and **5** (Scheme 1) through an attack to either the most available *i*-position or the most nucleophilic but sterically hindered α-position, respectively. Compounds **5** represent derivatives with two substituents at the sp^3^-carbon atoms which sometimes tend to rearrange into the corresponding norcaradiene derivatives [29–30]. Our reactions of anion **2** with electrophiles confirm this tendency – we always observed either a dynamic equilibrium or total rearrangement of **5** into **6** upon formation.

**Scheme 2:**
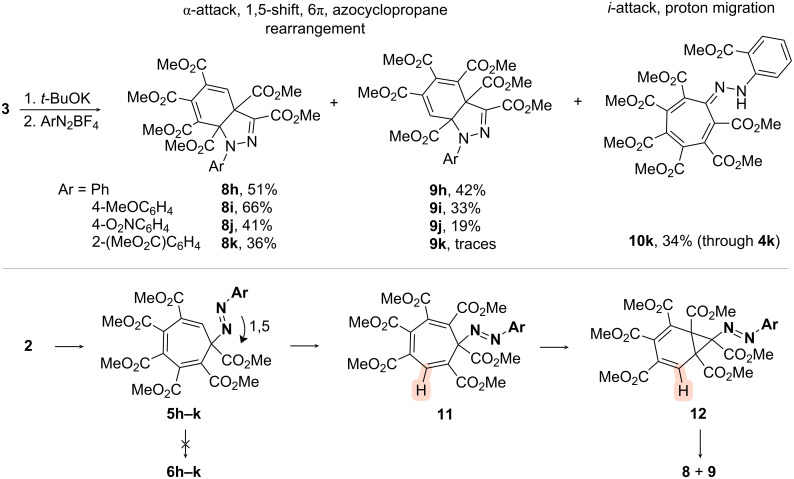
Reactions of anion **2** generated from cycloheptatriene **3** with diazonium salts.

Thus, halogenation of hexa(methoxycarbonyl)cycloheptatrienyl anion **2** formed from **3** led to different outcomes with chlorine, bromine and iodine (Scheme 1). Chlorination gave exclusively *i*-substituted symmetric cycloheptatriene **4a** in an excellent yield, the structure being confirmed using single crystal X-ray analysis (CCDC 2495984). Bromination afforded a mixture of symmetric cycloheptatriene **4b** and norcaradiene **6b**, while intermediate cycloheptatriene **5b** was not even observed. Iodination gave exclusively norcaradiene **6c**. Previously reported [31] halogenations of anion **1** only afforded the corresponding octasubstituted cycloheptatriene derivatives. The reactions of **2** with alkyl halides were selective in terms of the initial reaction – the *i*-substitution was never observed. However, the alkylation reactions were even more intricate than halogenation reactions. The reactions with methyl iodide and propargyl bromide resulted in equilibrium mixtures of α-alkylated cycloheptatrienes **5d**,**e** and the corresponding norcaradienes **6d**,**e**. At the same time, benzyl bromide afforded pure norcaradiene **6f** in a good yield. Apparently, the formation of a pure norcaradiene product in the case of benzylation, unlike in the close analogues, is due to a steric repulsion. The structure of norcaradiene **6f** was confirmed through single crystal X-ray analysis (CCDC 2495985). Allylation of anion **2** was followed by intramolecular [4 + 2]-cycloaddition in norcaradiene **6g** to form caged tetracyclodecene **7g** similar to that previously obtained from anion **1** [17] and also confirmed through single crystal X-ray analysis (CCDC 2496140). Using aryldiazonium salts as electrophiles afforded an even more complicated cascade process. Initial reactions with phenyl- or 4-substituted aryldiazonia involved electrophilic attacks onto the α-position. The azo compounds formed do not undergo a direct electrocyclization into norcaradienes **6h–k** but through a preliminary 1,5-arylazo shift which proceeds in a single direction of the seven-membered ring (clockwise for the structure in Scheme 2) into **11**. The 1,5-arylazo shift has been previously investigated in cyclopentadiene systems [32]. Subsequent formation of norcaradienes **12** and azocyclopropane rearrangement [33–35] afford a mixture of isomeric dihydroindazole derivatives **8** and **9** which differ in positioning of one ester group. The sterically hindered 2-(methoxycarbonyl)phenyldiazonium ion afforded only one isomer of dihydroindazole **8k** which indicates the influence of the steric factor on the azocyclopropane rearrangement. However, the reaction additionally gave the product of an *i*-substitution with subsequent proton migration to form hydrazonocycloheptatriene **10k**. Notably, dihydroindazoles **8** and **9** are similar to that previously formed from **1** [17].

In this investigation the most questionable is the attack onto the *i*-position. In our previous studies we have substantially investigated the geometry of anion **2** in the most stable conformation, which has a partial positive charge on the *i-*positioned carbon atom [36], confirmed by an unusually downfield shifted signal of the CH fragment in the ^13^C NMR spectrum (δ 160.4 ppm in CD_3_CN) [21]. Moreover, anion **2** underwent a nucleophilic attack onto the *i*-position by amines to form fluorescent 5-hydroxyisoquinolones [36–37]. Additionally, the analyses of both HOMO and Fukui *f*^–^ function shows little or no nucleophilicity at this position (Figure 2). Therefore, the formation of products **4a**,**b** and **10k** could proceed through the intermediary formation of the corresponding compounds **5** and their subsequent isomerization. However, these isomerizations are known to proceed as 1,5-sigmatropic shift [32] as observed in the formation of compounds **8** and **9**, whereas the formation of products **4** through this pathway would require at least two consequent shifts. Thus, the absence of any intermediate-shift products along with the formation of **4a**,**b**, as well as the formation of hydrazone product **10k** exclusively in the case of the most sterically hindered diazonium electrophile leads to the conclusion that the direct electrophilic attack onto the *i*-position could take place in all these cases.

**Figure 3:**
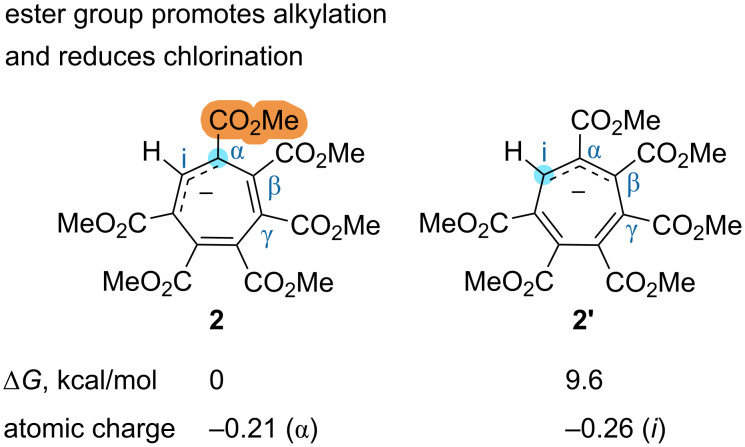
Two conformers of hexa(methoxycarbonyl)cycloheptatrienyl anion **2** and **2'**. The energies were obtained at the revDSD-PBEP86(D4)/aug-ccpVTZ/CPCM(acetonitrile)//r^2^SCAN-3c/CPCM(acetonitrile) level of theory.

To clarify this, we analyzed the set of conformations of anion **2** and found that conformer **2'**, with an allyl-anionic fragment shifted to form a nucleophilic CH fragment (Figure 3), is only 9.6 kcal/mol less stable than the main conformer **2** (the energy values of other conformers are given in Supporting Information File 1). Note, that in Figure 3, the two shortest bonds are depicted as double bonds, the three longest as single bonds, and the intermediary bonds are indicated as tautomeric. Therefore, if a reaction barrier for the α-attack (formation of compounds **5**) is above 9.6 kcal/mol, the reaction obeys the Curtin–Hammett principle [38]. At least, the reaction times and conditions required for the alkylation reactions investigated, which proceed through an α-attack, indicate relatively high barriers. This in turn indicates that the selectivity observed in alkylation reactions (α-attack) is due to some stabilization of the transition states with an adjacent ester group, not to the stability of the main conformer. Conversely, the reactions with halogens and diazonium salts are immediate at room temperature and the α-selectivity, when observed, can be due to the stability of the main conformer. The transition from iodine to chlorine is associated with an increase in energy of the lowest unoccupied molecular orbital of halogen molecules and a decrease in their electrophilicity. Therefore, if an electrophilic mechanism takes place, iodine apparently reacts with the main conformer faster than a transition to conformer **2'** may occur. In the case of bromine, the two processes demonstrate similar rates. The formation of product **4a** upon chlorination indicates the reaction barrier above 9.6 kcal/mol. Therefore, unlike with alkylation, the ester group adjacent to the α-position reduces chlorination of the main conformer, while hydrogen, conversely, induces the *i*-chlorination. Besides the obvious steric influence, the ester group slightly reduces the negative charge at the nucleophilic center (Figure 3) and can also influence the electronegative transition state in terms of orbital overlap.

**Scheme 3:**
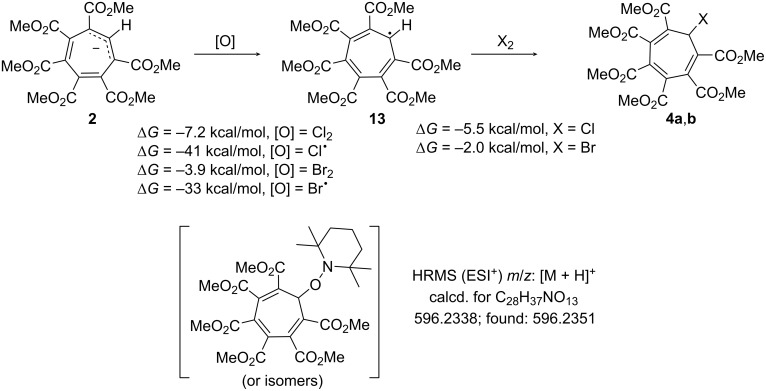
Radical mechanism for reactions of anion **2** with halogens, suggested structure of trapped product. The energies were obtained at the revDSD-PBEP86(D4)/aug-ccpVTZ/CPCM(acetonitrile)//r^2^SCAN-3c/CPCM(acetonitrile) level of theory.

Additionally, we considered the possibility of *i*-halogenation via a chain radical mechanism (Scheme 3). The initiation stage includes an oxidation of anion **2** into the corresponding radical **13** which in turn reacts with halogens to form products **4a**,**b** and a halogen atom which can also oxidize the anion. Quantum chemical calculations revealed negative free energy changes for all the stages of this reaction. A chlorination experiment with 2,2,6,6-tetramethylpiperidinyloxyl revealed a trace amount of a trap product detected by high-resolution mass spectrometry to support the radical mechanism. However, this does not exclude the possibility of a direct *i*-substitution.

## Conclusion

Umpolung reactivity of electron-deficient hexa(methoxycarbonyl)cycloheptatriene through its antiaromatic anion allows to introduce a connection between the cycloheptatriene core and an electrophilic reagent. The cycloheptatrienyl anion mainly exists as a conformer with the most nucleophilic center at the vicinal position to the hydrogen atom, however, the presence of other isomers allows to install a connection at the most sterically available CH fragment. The vicinal substitution tends to afford a ring-contraction into a corresponding norcaradiene derivative with a further transformation in the case of allylation. Reactions with diazonium salts mainly involve a vicinal addition of the reagent to the anion and subsequent 1,5-sigmatropic shift, 6π-electrocyclization, and azocyclopropane rearrangement into dihydroindazole derivatives. Analysis of the reaction conditions and selectivity revealed different controls in the cases of alkylation and halogenation reactions. Additionally, the reactions can be distinguished as driven by the relief of antiaromaticity.

## Supporting Information

File 1 Experimental procedures, product characterization, quantum chemical calculation details and copies of NMR spectra.

## Data Availability

All data that supports the findings of this study is available in the published article and/or the supporting information of this article.
