# Supplementary material for: Reactivity umpolung of the cycloheptatriene core in hexa(methoxycarbonyl)cycloheptatriene
Source: Beilstein J Org Chem. 2026 Jan 5;22:64–70. doi: 10.3762/bjoc.22.2 (PMC12794659; doi:10.3762/bjoc.22.2)
Supplement: File 1 — Experimental procedures, product characterization, quantum chemical calculation details and copies of NMR spectra. [file Beilstein_J_Org_Chem-22-64-s001.pdf]

BEILSTEIN JOURNAL OF ORGANIC CHEMISTRY

Supporting Information

for

Reactivity umpolung of the cycloheptatriene core in hexa(methoxycarbonyl)cycloheptatriene

Dmitry N. Platonov, Alexander Yu. Belyy, Rinat F. Salikov, Kirill S. Erokhin and Yury V. Tomilov

Beilstein J. Org. Chem. **2026,** *22,* 64–70. doi:10.3762/bjoc.22.2

Experimental procedures, product characterization, quantum chemical calculation details and copies of NMR spectra

License and Terms: This is a supporting information file under the terms of the Creative Commons Attribution License (https://creativecommons.org/licenses/by/4.0). Please note that the reuse, redistribution and reproduction in particular requires that the author(s) and source are credited and that individual graphics may be subject to special legal provisions.
The license is subject to the *Beilstein Journal of Organic Chemistry* terms and conditions: (https://www.beilstein-journals.org/bjoc/terms)

Table of Contents

1. General information S2
2. Experimental procedures S3
3. Computational data S7
4. Single crystal X-ray data S8
5. Spectral data S11
6. References S39

1. General information

Materials

Unless noted otherwise, all reagents were obtained from commercial suppliers and used without additional purification. Hexa(methoxycarbonyl)cycloheptatriene (**3**) [1], phenyldiazonium tetrafluoroborate [2], 4-methoxyphenyldiazonium tetrafluoroborate [2], 4-nitrophenyldiazonium tetrafluoroborate [3] and 2-methoxycarbonylphenyldiazonium tetrafluoroborate [3] were obtained using literature procedures. All reagents and solvents are commercially available and used without additional purification.

Instrumentations

NMR spectroscopy: ^{1}H NMR and ^{13}C NMR spectra were recorded on a Bruker AM-300 spectrometer (^{1}H: 300 MHz, ^{13}C: 75 MHz). Chemical shifts are reported in parts per million (ppm), using tetramethylsilane for $CDCl_3$ and residual solvent signal for DMSO-d_6 as internal standards. Multiplicities were given as: s (singlet), d (doublet), t (triplet), m (multiplets), dd (doublet of doublets), dt (doublet of triplets), and br (broad). Coupling constants (J) were recorded in hertz (Hz).

High-resolution mass spectrometry: HRMS spectra were obtained with a Bruker microTOF II instrument (ESI, positive or negative ion modes, capillary voltage 4500 V).

EI mass spectrometry: EIMS spectra were recorded with a Finnigan MAT INCOS 50.

Liquid chromatography: TLC was performed on pre-coated plates Merck Silica Gel 60 GF254. Flash chromatography was performed on silica gel Machery-Nagel 60 40–60 mesh.

2. Experimental procedures

General procedure for the halogenation reactions

To a solution of 250 mg (0.57 mmol) of hexa(methoxycarbonyl)cycloheptatriene **3** in 5 mL of CH_3CN was added 76 mg (0.68 mmol) of *t*-BuOK and the reaction mixture was stirred for 30 min. Then 0.63 mmol of the corresponding halogen (in case of Cl_2 it was blown through until the dark blue solution became colorless). The solvent was removed in vacuo, the residue was dissolved in ethyl acetate, washed with water and dried over Na_2SO_4. Then the solvent was removed in vacuo, and the product was recrystallized from ethyl acetate.

Hexamethyl 7-chlorocyclohepta-1,3,5-triene-1,2,3,4,5,6-hexacarboxylate ***(4a)*** was obtained using chlorine in a yield of 95%, white crystals, mp 112–115°C.

^{1}H NMR ($CDCl_3$, 300 MHz): δ [ppm] 6.22 (s, 1H, CH), 3.86 (s, 6H, 2OMe), 3.84 (s, 6H, 2OMe), 3.82 (s, 6H, 2OMe).

^{13}C NMR ($CDCl_3$, 75 MHz): δ [ppm] 164.8, 164.7, 165.5, 137.8, 135.7, 132.0, 53.7, 53.5, 53.3, 46.9

HRMS (ESI+) m/z: $[M + NH_4]^+$ Calcd. for $C_{19}H_{19}ClO_{12}$ 492.0903; Found: 492.0898.

Hexamethyl 7-bromobicyclo[4.1.0]hepta-2,4-diene-1,2,3,4,5,7-hexacarboxylate ***(6b)*** was obtained using bromine and purified by preparation of concentrated solution of the crude mixture in ethyl acetate and recrystallization at 4 °C to afford the product in a yield of 30%, white crystals, mp 181–184°C.

^{1}H NMR ($CDCl_3$, 300 MHz): δ [ppm] 3.90 (s, 1H, CH), 3.87 (s, 3H, OMe), 3.88 (s, 6H, 2OMe), 3.76 (s, 3H, OMe), 3.62 (s, 3H, OMe), 3.59 (s, 3H, OMe).

^{13}C NMR ($CDCl_3$, 75 MHz): δ [ppm] 167.1, 166.0, 165.9, 165.6, 165.1, 164.0, 132.4, 131.9, 130.5, 129.3, 53.1, 53.0, 53.0, 52.9, 52.7, 52.6, 39.1, 38.5, 33.6.

HRMS (ESI+) m/z: $[M + NH_4]^+$ Calcd. for $C_{19}H_{19}BrO_{12}$ 536.098; Found: 536.0405.

Hexamethyl 7-bromocyclohepta-1,3,5-triene-1,2,3,4,5,6-hexacarboxylate ***(4b)*** was obtained by concentration of the mother liquor from **6b** and crystallization at 4 °C to afford the product in a yield of 60%, white crystals, mp142–145°C.

^{1}H NMR ($CDCl_3$, 300 MHz): δ [ppm] 6.26 (s, 1H, CH), 3.87 (s, 6H, 2OMe), 3.85 (s, 6H, 2OMe), 3.82 (s, 6H, 2OMe).

^{13}C NMR ($CDCl_3$, 75 MHz): δ [ppm] 164.9, 164.7, 163.4, 138.7, 136.5, 134.3, 53.7, 53.6, 53.3, 34.7

HRMS (ESI+) m/z: $[M + NH_4]^+$ Calcd. for $C_{19}H_{19}BrO_{12}$ 536.0398; Found: 536.0389.

Hexamethyl 7-iodobicyclo[4.1.0]hepta-2,4-diene-1,2,3,4,5,7-hexacarboxylate ***(6c)*** was obtained using iodine in a yield of 59%, brownish crystals, mp 189–191°C.

^{1}H NMR ($CDCl_3$, 300 MHz): δ [ppm] 3.85 (s, 3H, OMe), 3.83 (s, 3H, OMe), 3.90 (s, 1H, CH), 3.87 (s, 3H, OMe), 3.78 (s, 6H, 2OMe), 3.76 (s, 3H, OMe), 3.62 (s, 3H, OMe), 3.59 (s, 3H, OMe).

^{13}C NMR ($CDCl_3$, 75 MHz): δ [ppm] 167.1, 166.0, 165.9, 165.6, 165.2, 164.0, 132.4, 131.9, 130.5, 129.3, 53.1, 53.0, 53.0, 52.9, 52.7, 52.6, 39.0, 38.5, 33.6.

Experiments on obtaining ESI HRMS as well as EI MS spectra gave no results.

General procedure for the alkylation reactions

To a solution of 250 mg (0.57 mmol) of hexa(methoxycarbonyl)cycloheptatriene (**3**) in 5 mL CH_3CN was added 76 mg (0.68 mmol) of *t*-BuOK and the reaction mixture was stirred for 30 min. Then 0.63 mmol of the corresponding alkyl halide was added and the reaction mixture was heated at 60 °C for the appropriate time (30 h with methyl iodide and propargyl bromide, 20 h with benzyl bromide). The solvent was removed in vacuo, and the residue was purified by column chromatography on silica gel (chloroform/ethyl acetate 4:1) to afford the product.

*An equilibrium mixture of hexamethyl 1-methylcyclohepta-2,4,6-triene-1,2,3,4,5,6-hexacarboxylate (**5d**) and hexamethyl 7-methylbicyclo[4.1.0]hepta-2,4-diene-1,2,3,4,5,7-hexacarboxylate (**6d**) (14:86)* was obtained using methyl iodide in a yield of 73%, colorless oil °C. A ^{1}H NMR allowed to distinguish the signals of both compounds, a ^{13}C NMR did not. The ratio was determined by integration of signals of methyl groups.

*Hexamethyl 1-methylcyclohepta-2,4,6-triene-1,2,3,4,5,6-hexacarboxylate (**5d**)*

^{1}H NMR (300 MHz, $CDCl_3$) δ [ppm] 7.98 (s, 1H, CH), 3.80-3.40 (6x s, 18H, OMe), 1.58 (s, 3H, Me).

*Hexamethyl 7-methylbicyclo[4.1.0]hepta-2,4-diene-1,2,3,4,5,7-hexacarboxylate (**6d**)*

^{1}H NMR (300 MHz, $CDCl_3$) δ [ppm] 3.78 (s, 3H, OMe), 3.75 (s, 6H, OMe), 3.74 (s, 3H, OMe), 3.71 (s, 3H, OMe), 3.69 (s, 1H, CH), 3.59 (s, 3H, OMe), 1.00 (s, 3H, Me).

*An equilibrium mixture of **5d** and **6d***

^{13}C NMR (75 MHz, $CDCl_3$) δ [ppm] 171.3, 170.4, 167.1, 166.6, 165.7, 165.4, 165.1, 165.0, 164.9, 164.4, 164.0, 163.8, 145.9, 140.8, 138.5, 135.1, 133.9, 133.0, 132.6, 132.0, 131.1, 130.1, 128.4, 127.8, 127.2, 65.7, 53.1, 53.0, 52.9, 52.9, 52.8, 52.7, 52.6, 49.1, 40.8, 37.0, 22.6, 19.5, 15.1, 10.4.

HRMS (ESI+) m/z: $[M + NH_4]^+$ Calcd. for $C_{20}H_{22}O_{12}$ 472.1450; Found: 472.1462.

*An equilibrium mixture of hexamethyl 1-(prop-2-yn-1-yl)cyclohepta-2,4,6-triene-1,2,3,4,5,6-hexacarboxylate (**5e**) and hexamethyl 7-(prop-2-yn-1-yl)bicyclo[4.1.0]hepta-2,4-diene-1,2,3,4,5,7-hexacarboxylate (**6e**) (23:77) was obtained using propargyl bromide in a yield of 96%, colorless oil. The ratio was determined by integration of signals of alkyne CH-groups.*

^{1}H NMR (300 MHz, $CDCl_3$) δ [ppm] 7.32 (s, CH), 4.16 – 3.49 (m, 12H), 3.32 – 3.02 (m), 2.84 (dd, J = 17.6, 2.4 Hz, 2H), 2.16 (br. s, CH), 1.99 (t, J = 2.4 Hz, 1H), 1.76 (dd, J = 17.6, 2.4 Hz).

^{13}C NMR (75 MHz, $CDCl_3$) δ [ppm] 169.5, 167.8, 166.7, 165.6, 165.2, 164.7, 164.4, 137,0, 133.9, 133.3, 132.9, 131.9, 131.4, 129.7, 128.8, 78.5, 70.8, 53.4, 53.3, 53.1, 53.1, 53.0, 52.9, 43.9, 42.2, 40.6, 35.8, 26.1, 15.8.

HRMS (ESI+) m/z: $[M +NH_4]^+$ Calcd. for $C_{22}H_{22}O_{12}$ 496.1450; Found: 496.1464

*Hexamethyl 7-benzylbicyclo[4.1.0]hepta-2,4-diene-1,2,3,4,5,7-hexacarboxylate (**6f**)* was obtained using benzyl bromide in a yield of 82%, white crystals, mp 128–131°C.

^{1}H NMR ($CDCl_3$, 300 MHz): δ [ppm] 7.12-7.25 (m, 3H, Ph), 7.00-7.14 (m, 2H, Ph), 3.85 (s, 3H, OMe), 3.83 (s, 3H, OMe), 3.81 (s, 3H, OMe), 3.79 (s, 1H, CH), 3.76 (s, 3H, OMe), 3.72 (s, 3H, OMe), 3.67 (s, 3H, OMe), 3.30 (d, 1H, J=5.0Hz, CH2), 2.23 (d, 1H, J=5.0Hz, CH_2).

^{13}C NMR ($CDCl_3$, 75 MHz): δ [ppm] 169.9, 167.5, 165.8, 165.6, 164.9, 164.8, 136.3, 133.4, 133.1, 130.1, 129.0, 128.3, 126.9, 53.1, 53.1, 53.1, 53.0, 53.0, 39.2, 36.1, 31.3, 28.7.

HRMS (ESI+) m/z: $[M + NH_4]^+$ Calcd. for $C_{26}H_{26}O_{12}$ 548.1763; Found: 548.1768.

Synthesis of hexamethyl 1*H*-1,3-methanocyclopropa[cd]indene-2a,2a1,3,4,5,5a(2*H*,2b*H*)-hexacarboxylate (7g).

To a solution of 100 mg (0.23 mmol) of hexa(methoxycarbonyl)cycloheptatriene (**3**) in 2 mL CH_3CN in a sealed vial was added 52 mg (0.46 mmol) of *t*-BuOK and the reaction mixture was stirred for 30 min. Then 0.12 mL (1.36 mmol) of allyl bromide were added and the reaction mixture was heated at 80 °C for 36 h The solvent was removed in vacuo, and the residue was purified by column chromatography on silica gel (toluene/ethyl acetate 4:1) and additionally recrystallized from methanol to afford the product in a yield of 55%, white crystals, mp 132–134°C.

^{1}H NMR (300 MHz, $CDCl_3$) δ [ppm] 3.82 (s, 3H), 3.82 (s, 3H), 3.81 (s, 3H), 3.77 (s, 3H), 3.76 (s, 3H), 3.69 (s, 3H), 3.02 (s, 1H), 2.88 (dd, J = 12.2, 5.3 Hz, 1H), 2.42 (dd, J = 7.7, 5.2 Hz, 1H), 2.02 (d, J = 12.1 Hz, 1H), 1.89 (d, J = 12.4 Hz, 1H), 1.69 – 1.62 (m, 1H).

^{13}C NMR (75 MHz, $CDCl_3$) δ [ppm] 171.8, 170.1, 168.5, 167.7, 165.3, 164.2, 144.2, 139.8, 59.3, 52.9, 52.9, 52.6, 52.5, 52.5, 52.4, 49.9, 49.5, 43.4, 39.2, 36.3, 35.9, 35.7.

HRMS (ESI+) m/z: $[M + Na]^+$ Calcd. for $C_{22}H_{24}O_{12}$ 503.1160; Found: 548.1768.

General procedure for the reactions of anion 2 with aryldiazonium salts

To a solution of 250 mg (0.57 mmol) of hexa(methoxycarbonyl)cycloheptatriene (**3**) in 5 mL CH_3CN was added 76 mg (0.68 mmol) of *t*-BuOK and the reaction mixture was stirred for 30 min. Then 0.63 mmol of the corresponding diazonium tetrafluoroborate was added and the reaction mixture was stirred at room temperature for another hour. The solvent was removed in vacuo, the residue was purified by column chromatography on silica gel (chloroform/ethyl acetate 4:1) to afford the products.

Hexamethyl 1-phenyl-1H-indazole-3,3a,5,6,7,7a-hexacarboxylate ***(8h)*** was obtained using phenyldiazonium tetrafluoroborate in a yield of 51%, yellow non-crystalline solid, mp 165–166°C.

^1H NMR (DMSO-d_6, 300 MHz): δ [ppm] 7.25-7.40 (m, 5H, Ph), 7.00 (s, 1H, =CH-), 3.80 (s, 6H, 2OMe), 3.78 (s, 3H, OMe), 3.72 (s, 3H, OMe), 3.53 (s, 3H, OMe), 3.34 (s, 3H, OMe).

^{13}C NMR (DMSO-d_6, 75 MHz): δ [ppm] 166.5, 166.4, 166.3, 164.2, 163.6, 161.6, 141.2, 138.2, 133.7, 133.2, 128.8, 127.9, 127.3, 126.6, 123.5, 79.8, 66.5, 54.7, 54.0, 53.6, 53.5, 53.1, 53.1.

HRMS (ESI+) m/z: $[M +H]^+$ Calcd. for $C_{23}H_{22}N_2O_{10}$ 545.1402; Found: 545.1399.

Hexamethyl 1-phenyl-1H-indazole-3,3a,4,5,6,7a-hexacarboxylate ***(9h)*** was obtained using phenyldiazonium tetrafluoroborate in a yield of 42%, white crystals, mp 128–131°C.

^1H NMR ($CDCl_3$, 300 MHz): δ [ppm] 7.11-7.35 (m, 5H, Ph), 7.08 (s, 1H, =CH-), 3.85 (s, 3H, OMe), 3.84 (s, 3H, OMe), 3.78 (s, 6H, 2OMe), 3.77 (s, 1H, CH), 3.73 (s, 3H, OMe).

^{13}C NMR ($CDCl_3$, 75 MHz): δ [ppm] 167.2, 166.5, 165.8, 164.9, 164.0, 161.3, 141.0, 136.2, 133.8, 130.8, 129.6, 129.5, 129.4, 124.8, 119.1, 79.8, 66.5, 54.7, 54.0, 53.6, 53.5, 53.1, 53.1.

HRMS (ESI+) m/z: $[M + NH_4]^+$ Calcd. for $C_{23}H_{22}N_2O_{10}$ 545.1402; Found: 545.1798.

Hexamethyl 1-(4-methoxyphenyl)-1H-indazole-3,3a,5,6,7,7a-hexacarboxylate (***8i)*** was obtained using 4-methoxyphenyldiazonium tetrafluoroborate in a yield of 66%, yellow crystals, mp 162–165°C.

^1H NMR ($CDCl_3$, 300 MHz): δ [ppm] 7.33-7.38 (m, 2H, C_6H_4), 7.21 (s, 1H, =CH-), 6.79-6.85 (m, 2H, C_6H_4), 3.94 (s, 3H, OMe), 3.87 (s, 3H, OMe), 3.84 (s, 3H, OMe), 3.78 (s, 6H, 2OMe), 3.59 (s, 3H, OMe), 3.35 (s, 3H, OMe).

^{13}C NMR (CDCl3, 75 MHz): δ [ppm] 166.9, 166.6, 166.6, 163.9, 163.3, 161.9, 159.0, 138.0, 134.0, 133.7, 132.1, 128.9, 126.3, 122.4, 113.5, 79.7, 66.3, 55.4, 53.9, 53.1, 52.9, 52.8, 52.6, 52.4.

HRMS (ESI+) m/z: $[M +NH_4]^+$ Calcd. for $C_{24}H_{24}N_2O_{11}$ 592.1773; Found: 592.1771.

Hexamethyl 1-(4-methoxyphenyl)-1H-indazole-3,3a,4,5,6,7a-hexacarboxylate ***(9i)*** was obtained using 4-methoxyphenyldiazonium tetrafluoroborate in a yield of 33%, yellow non-crystalline solid.

^1H NMR ($CDCl_3$, 300 MHz): δ [ppm] 7.14-7.18 (m, 2H, C_6H_4), 6.95 (s, 1H, =CH-), 6.83-6.87 (m, 2H, C_6H_4), 3.84 (s, 3H, OMe), 3.81 (s, 3H, OMe), 3.78 (s, 6H, 2OMe), 3.77 (s, H, OMe), 3.76 (s, 3H, OMe), 3.71 (s, 3H, OMe).

^{13}C NMR (126 MHz, DMSO-d6): δ [ppm] 167.0, 166.5, 165.8, 164.8, 164,0, 161.3, 157.7, 135.5, 134.2, 130.7, 129.7, 129.3, 128.5, 126.7, 123.2, 114.4, 113.3, 80.4, 55.5, 53.9, 53.8, 52.9, 52.9, 52.8, 52.5.

HRMS (ESI+) m/z: $[M +NH_4]$+ Calcd. for $C_{24}H_{24}N_2O_{11}$ 592.1773; Found: 592.1775.

Hexamethyl 1-(4-nitroxyphenyl)-1H-indazole-3,3a,5,6,7,7a-hexacarboxylate ***(8j)*** was obtained using 4-nitrophenyldiazonium tetrafluoroborate in a yield of 41%, orange non-crystalline solid.

^{1}H NMR ($CDCl_3$, 300 MHz): δ [ppm] 8.17 (d, 2H, J=9.0 Hz, C_6H_4), 7.56 (d, 2H, J=9.0 Hz, C_6H_4), 7.19 (s, 1H, =CH-), 3.96 (s, 3H, OMe), 3.93 (s, 3H, OMe), 3.85 (s, 3H, OMe), 3.80 (s, 3H, OMe), 3.58 (s, 3H, OMe), 3.53 (s, 3H, OMe).

^{13}C NMR (CDCl3, 75 MHz): δ [ppm] 166.5, 166.2, 165.7, 164.4, 163.0, 161.4, 147.1, 145.8, 139.1, 136.3, 133.4, 127.2, 126.1, 123.7, 123.0, 79.0, 67.0, 54.2, 53.5, 53.2, 53.0, 53.0 52.8.

HRMS (ESI+) m/z: $[M+H]^+$ Calcd. for $C_{23}H_{21}N_3O_{12}$ 590.1253; Found: 590.1254.

Hexamethyl 1-(4-nitroxyphenyl)-1H-indazole-3,3a,4,5,6,7a-hexacarboxylate (***9j***) was obtained using 4-niotrophenyldiazonium tetrafluoroborate in a yield of 19%, orange non-crystalline solid.

^{1}H NMR ($CDCl_3$, 300 MHz): δ [ppm] 8.22-827 (m, 2H, C6H4), 7.25-7.30 (m, 2H, C6H4), 7.11 (s, 1H, =CH-), 3.91 (s, 3H, OMe), 3.84 (s, 3H, OMe), 3.83 (s, 3H, OMe), 3.82 (s, 3H, OMe), 3.80 (s, 6H, 2OMe).

^{13}C NMR ($CDCl_3$, 75 MHz): δ [ppm] 166.5, 166.1, 165.7, 164.6, 163.6, 160.7, 146.0, 143.2, 139.4, 131.0, 130.4, 128.1, 125.5, 116.5, 114.1, 78.3, 66.7, 54.6, 54.2, 53.2, 53.1, 53.0 52.7.

HRMS (ESI+) m/z: $[M+NH_4]^+$ Calcd. for $C_{23}H_{21}N_3O_{12}$ 607.1518; Found: 607.1518.

Hexamethyl 1-(2-(methoxycarbonyl)phenyl)-1H-indazole-3,3a,5,6,7,7a-hexacarboxylate (***8k***) was obtained using 2-methoxycarbonylphenyldiazonium tetrafluoroborate in a yield of 36%, yellow solid, mp 150–151°C.

^{1}H NMR ($CDCl_3$, 300 MHz): δ [ppm] 8.16 (d, 1H, J=8.0 Hz, C6H4), 7.91 (d, 1H, J=8.0 Hz, C_6H_4), 7.59 (t, 1H, J=8.0 Hz, C6H4), 7.42 (t, 1H, J=8.0 Hz, C_6H_4), 7.38 (s, 1H, =CH-), 3.85 (s, 6H, 2OMe), 3.82 (s, 3H, OMe), 3.80 (s, 3H, OMe), 3.73 (s, 3H, OMe), 3.65 (s, 3H, OMe), 3.20 (s, 3H, OMe).

^{13}C NMR ($CDCl_3$, 75 MHz): δ [ppm] 167.6, 167.5, 166.6, 165.0, 163.9, 163.7, 162,0, 139.2, 138.9, 133.9, 133.4, 132.7, 131.7, 131.1, 130.4, 129.1, 125.9, 120.5, 79.7, 66.3, 54.0, 53.3, 52.7, 52.6, 52.4 52.3, 52.1.

HRMS (ESI+) m/z: $[M+H]^+$ Calcd. for $C_{25}H_{24}N_2O_{12}$ 603.1457; Found: 603.1461.

Hexamethyl 7-(2-(2-(methoxycarbonyl)phenyl)hydrazineylidene)cyclohepta-1,3,5-triene-1,2,3,4,5,6-hexacarboxylate (**10k**) was obtained using 2-methoxycarbonylphenyldiazonium tetrafluoroborate in a yield of 34%, orange non-crystalline solid.

^{1}H NMR ($CDCl_3$, 300 MHz): δ [ppm] 11.03 (s, 1H, NH), 7.86 (d, 2H, J=8.6 Hz, C_6H_4), 7.49 (t, 1H, J=8.6 Hz, C_6H_4), 6.94 (t, 1H, J=8.6 Hz, C_6H_4), 3.93 (s, 3H, OMe), 3.87 (s, 6H, 2OMe), 3.83 (s, 3H, OMe), 3.73 (s, 6H, 2OMe), 3.71 (s, 3H, OMe).

^{13}C NMR ($CDCl_3$, 75 MHz): δ [ppm] 167.6, 165.6, 165.4, 164.4, 163.3, 144.9, 135.3, 134.7, 134.5, 133.8, 132.9, 131.9, 131.0, 121.1, 114.7, 112.8, 53.5, 53.5 (2C), 53.4 (2C), 52.6, 52.2.

HRMS (ESI+) m/z: $[M+H]^+$ Calcd. for $C_{25}H_{24}N_2O_{12}$ 603.1457; Found: 603.1454.

3. Computational data

Quantum chemical calculations were performed using ORCA 6.1 [4]. The CREST package identified the most stable conformers, for conformationally flexible species [5]. Geometries were optimized and frequencies were calculated at the r^2SCAN-3c/CPCM(acetonitrile) level, while single-point energies were obtained at the revDSD-PBEP86(D4)/aug-ccpVTZ/CPCM(acetonitrile) level [6]. Fukui functions f^- calculated in the Multiwfn 3.8 program package [7]. The partial charges of the atoms were calculated in the IboView [8] software using the Effective Oxidation States [EOS] method [9]. Visualization was performed with IboView and IQmol program package.

In all cases where energetic parameters were considered, the following expression for Gibbs free energy of formation was used:

$$\Delta G_f(X) = \Delta G_f(\text{r2SCAN} - \text{3c/CPCM(acetonitrile)}) - E_{SPE}(\text{r2SCAN} - \text{3c/CPCM(acetonitrile)}) + E_{SPE}(\text{revDSD} - \text{PBEP86(D4)/aug} - \text{ccpVTZ/CPCM(acetonitrile)}).$$

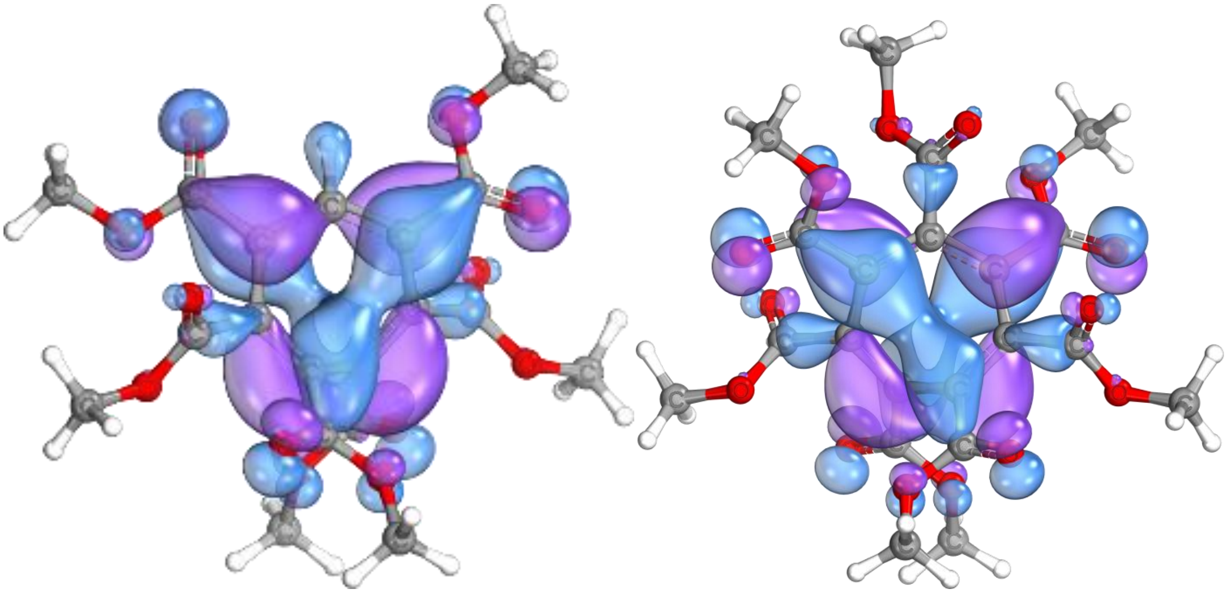

Figure S1: Highest occupied molecular orbitals of anions **2** and **1**.

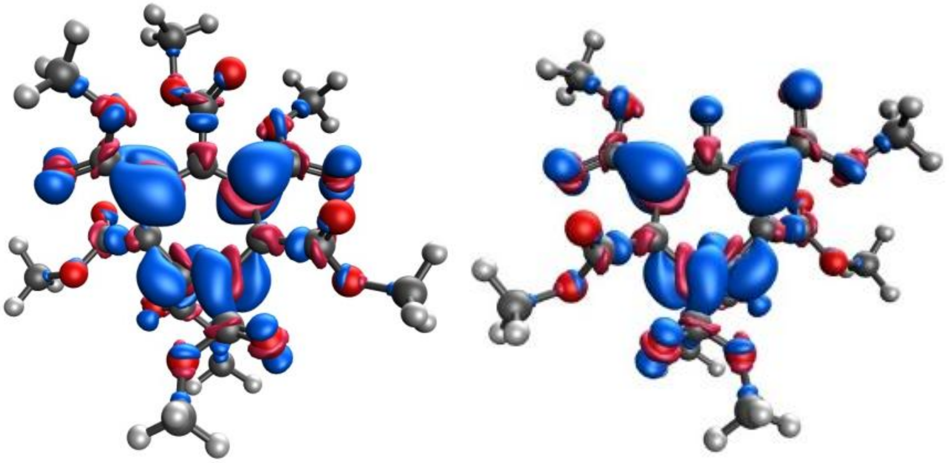

Figure S2: Fukui f^- function representations of anions **2** and **1**.

4. Single crystal X-ray data

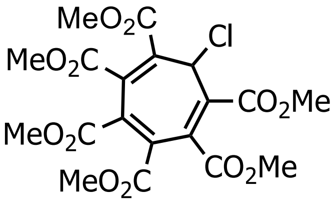

4a

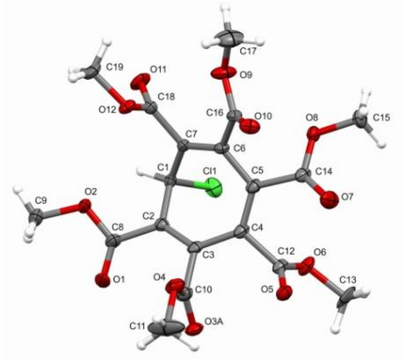

CCDC 2495984

Figure S3. ORTEP plot of the crystal structure of **4a**. Displacement ellipsoids for non-hydrogen atoms are shown at *p* = 50%.

Identification code	sci2536-1
Empirical formula	C19 H19 Cl O12
Formula weight	474.79
Temperature	100.0(2) K
Wavelength	1.54184 Å
Crystal system	Monoclinic
Space group	Cc
Unit cell dimensions	a = 8.37810(10) Å<br>b = 20.2601(2) Å<br>c = 12.85380(10) Å
Volume	2158.66(4) $Å^3$
Z	4
Density (calculated)	1.461 g/cm^3
Absorption coefficient	2.151 mm^{-1}
F(000)	984
Crystal size	0.44 x 0.23 x 0.07 mm^3
Theta range for data collection	4.365 to 80.715°.
Index ranges	-10<=h<=10, -25<=k<=25, -16<=l<=16
Reflections collected	23741
Independent reflections	3829 [R(int) = 0.0474]
Observed reflections	3821
Completeness to theta = 67.684°	100.0 %
Absorption correction	Gaussian
Max. and min. transmission	1.000 and 0.312
Refinement method	Full-matrix least-squares on F^2
Data / restraints / parameters	3829 / 30 / 335
Goodness-of-fit on F^2	1.120
Final R indices [I>2sigma(I)]	R1 = 0.0445, wR2 = 0.0943
R indices (all data)	R1 = 0.0446, wR2 = 0.0944
Absolute structure parameter	0.07(3)
Extinction coefficient	0.00071(7)
Largest diff. peak and hole	0.433 and -0.364 e.$Å^{-3}$

Table S1. X-ray crystallographic data of **2a**.

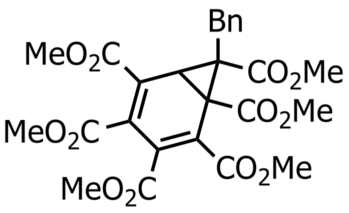

6f

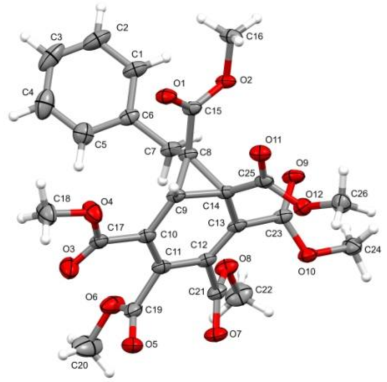

CCDC 2495985

Figure S4. ORTEP plot of the crystal structure of **6f**. Displacement ellipsoids for non-hydrogen atoms are shown at p = 50%.

Identification code	sci2539
Empirical formula	C26 H26 O12
Formula weight	530.47
Temperature	100.0(2) K
Wavelength	1.54184 Å
Crystal system	Monoclinic
Space group	$P2_1/c$
Unit cell dimensions	a = 10.65500(10) Å<br>b = 27.8851(3) Å<br>c = 10.52470(10) Å
Volume	2952.49(5) $Å^3$
Z	4
Density (calculated)	1.193 g/cm^3
Absorption coefficient	0.813 mm^{-1}
F(000)	1112
Crystal size	0.43 x 0.18 x 0.16 mm^3
Theta range for data collection	3.170 to 79.772°.
Index ranges	-13<=h<=13, -35<=k<=34, -12<=l<=13
Reflections collected	40060
Independent reflections	6380 [R(int) = 0.0358]
Observed reflections	5823
Completeness to theta = 67.684°	100.0 %
Absorption correction	Gaussian
Max. and min. transmission	1.000 and 0.541
Refinement method	Full-matrix least-squares on F^2
Data / restraints / parameters	6380 / 0 / 349
Goodness-of-fit on F^2	1.031
Final R indices [I>2sigma(I)]	R1 = 0.0408, wR2 = 0.1076
R indices (all data)	R1 = 0.0434, wR2 = 0.1095
Extinction coefficient	n/a
Largest diff. peak and hole	0.338 and -0.319 e.$Å^{-3}$

Table S2. X-ray crystallographic data of **6f**.

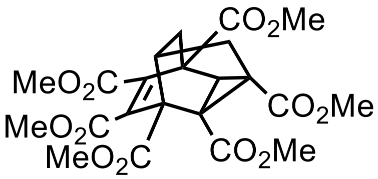

7g

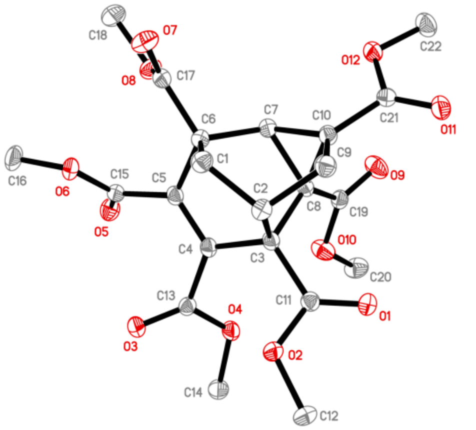

CCDC 2496140

Figure S5. ORTEP plot of the crystal structure of **7g**. Displacement ellipsoids for non-hydrogen atoms are shown at p = 50%.

Identification code	lit2232
Empirical formula	C22 H24 O12
Formula weight	480.41
Temperature	99.97(16) K
Wavelength	1.54184 Å
Crystal system	Monoclinic
Space group	C 1 2/c 1
Unit cell dimensions	a = 23.9236(3) Å<br>b = 10.64412(9) Å<br>c = 18.3599(2) Å
Volume	4347.17(8) $Å^3$
Z	8
Density (calculated)	1.468 g/cm^3
Absorption coefficient	1.038 mm^{-1}
F(000)	2016
Crystal size	0.15 x 0.12 x 0.08 mm^3
Theta range for data collection	3.975 to 77.862°.
Index ranges	-30<=h<=30, -13<=k<=13, -23<=l<=23
Reflections collected	8491
Independent reflections	8491 [R(int) = 0]
Observed reflections	7761
Completeness to theta = 67.684°	99.9 %
Absorption correction	Semi-empirical from equivalents
Max. and min. transmission	1.00000 and 0.79462
Refinement method	Full-matrix least-squares on F^2
Data / restraints / parameters	8491 / 0 / 315
Goodness-of-fit on F^2	1.089
Final R indices [I>2sigma(I)]	R1 = 0.0380, wR2 = 0.1061
R indices (all data)	R1 = 0.0406, wR2 = 0.1084
Extinction coefficient	0.00020(5)
Largest diff. peak and hole	0.359 and -0.249 e.$Å^{-3}$

Table S3. X-ray crystallographic data of **7g**.

5. Spectral data

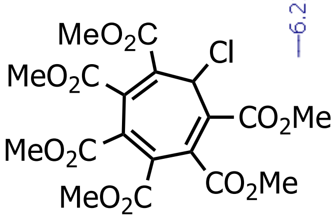

4a

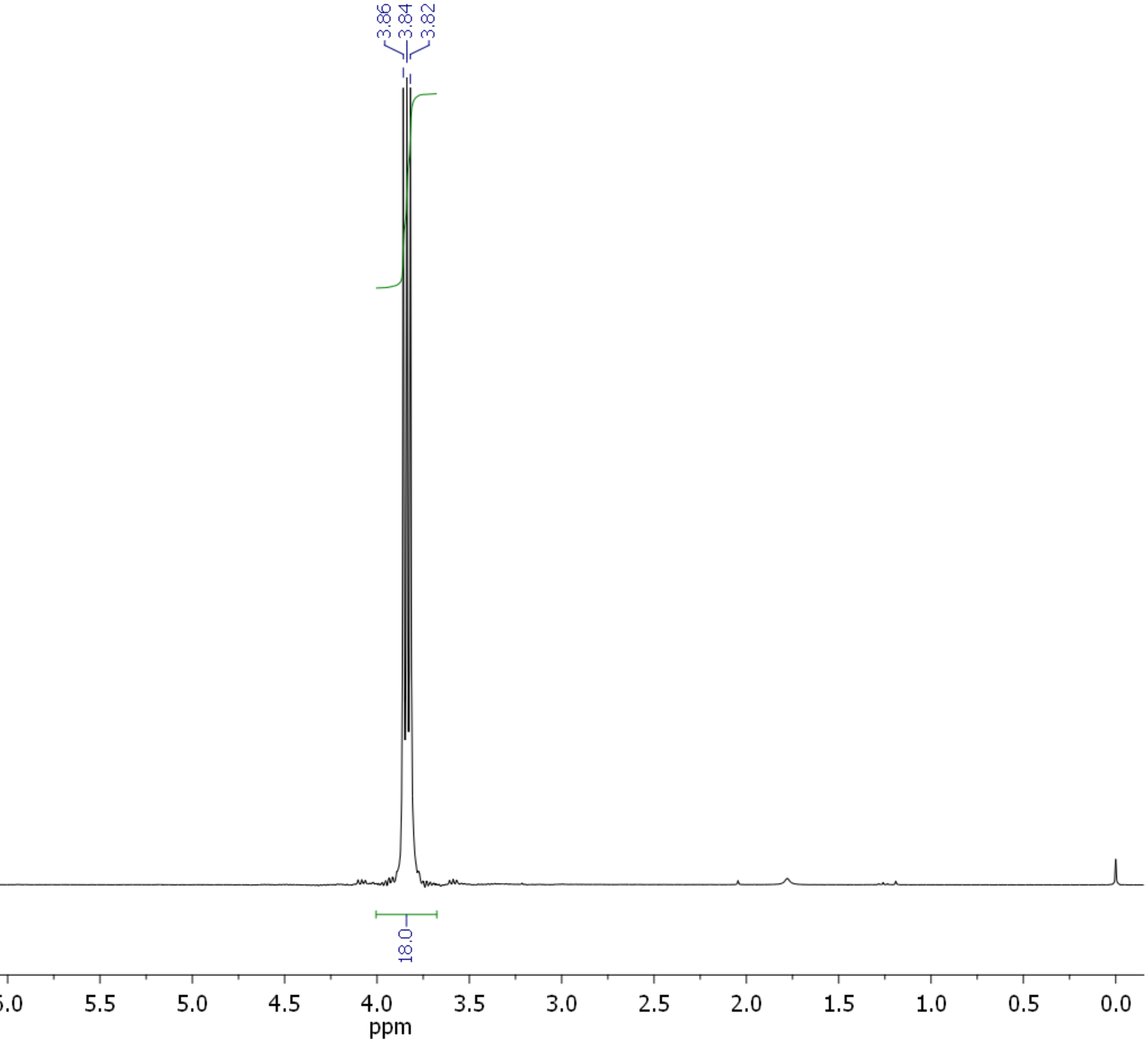

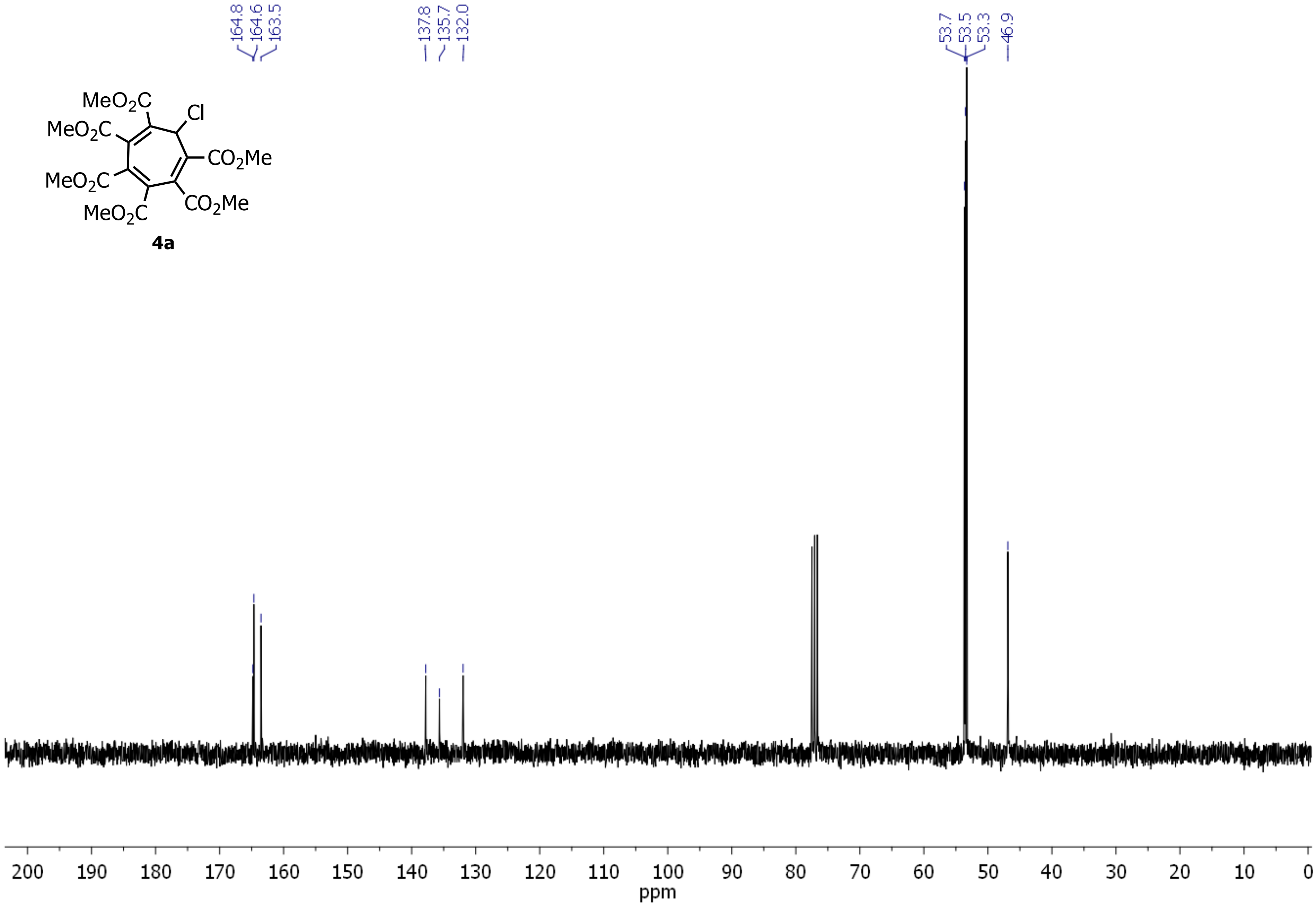
MeO2C
Cl
MeO2C
CO2Me
MeO2C
MeO2C
CO2Me
164.8
164.6
163.5
137.8
135.7
132.0
53.7
53.5
53.3
46.9
200 190 180 170 160 150 140 130 120 110 100 90 80 70 60 50 40 30 20 10 0
ppm

4a

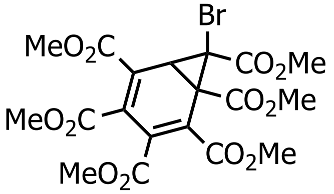

6b

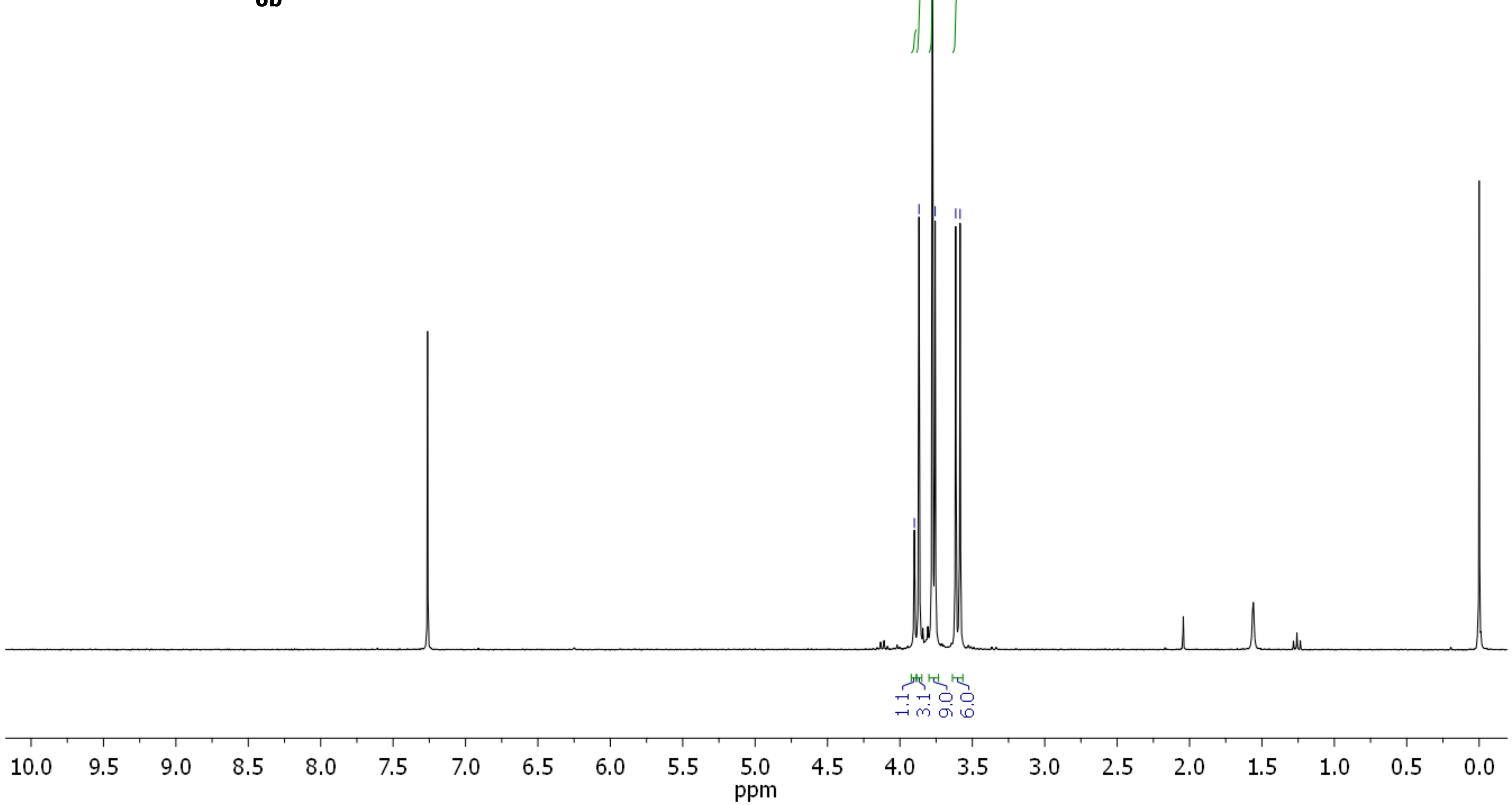
3.90
3.87
3.78
3.76
3.61
3.58
1.1
3.1
9.0
6.0
10.0
9.5
9.0
8.5
8.0
7.5
7.0
6.5
6.0
5.5
5.0
4.5
4.0
3.5
3.0
2.5
2.0
1.5
1.0
0.5
0.0
ppm

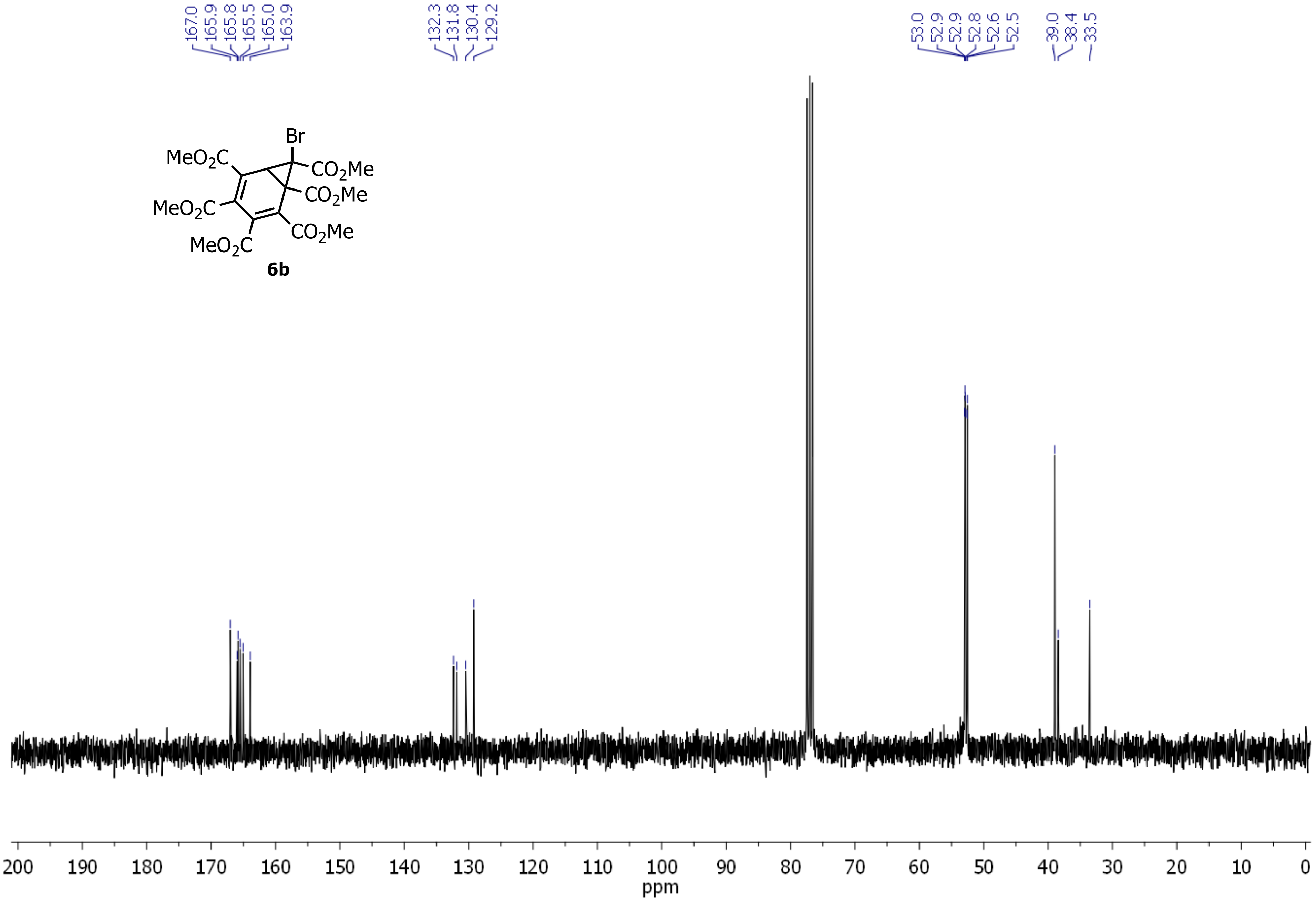

167.0
165.9
165.8
165.5
165.0
163.9
132.3
131.8
130.4
129.2
53.0
52.9
52.9
52.8
52.6
52.5
39.0
38.4
33.5
Br
MeO_2C
CO_2Me
CO_2Me
MeO_2C
CO_2Me
MeO_2C
6b
200 190 180 170 160 150 140 130 120 110 100 90 80 70 60 50 40 30 20 10 0
ppm

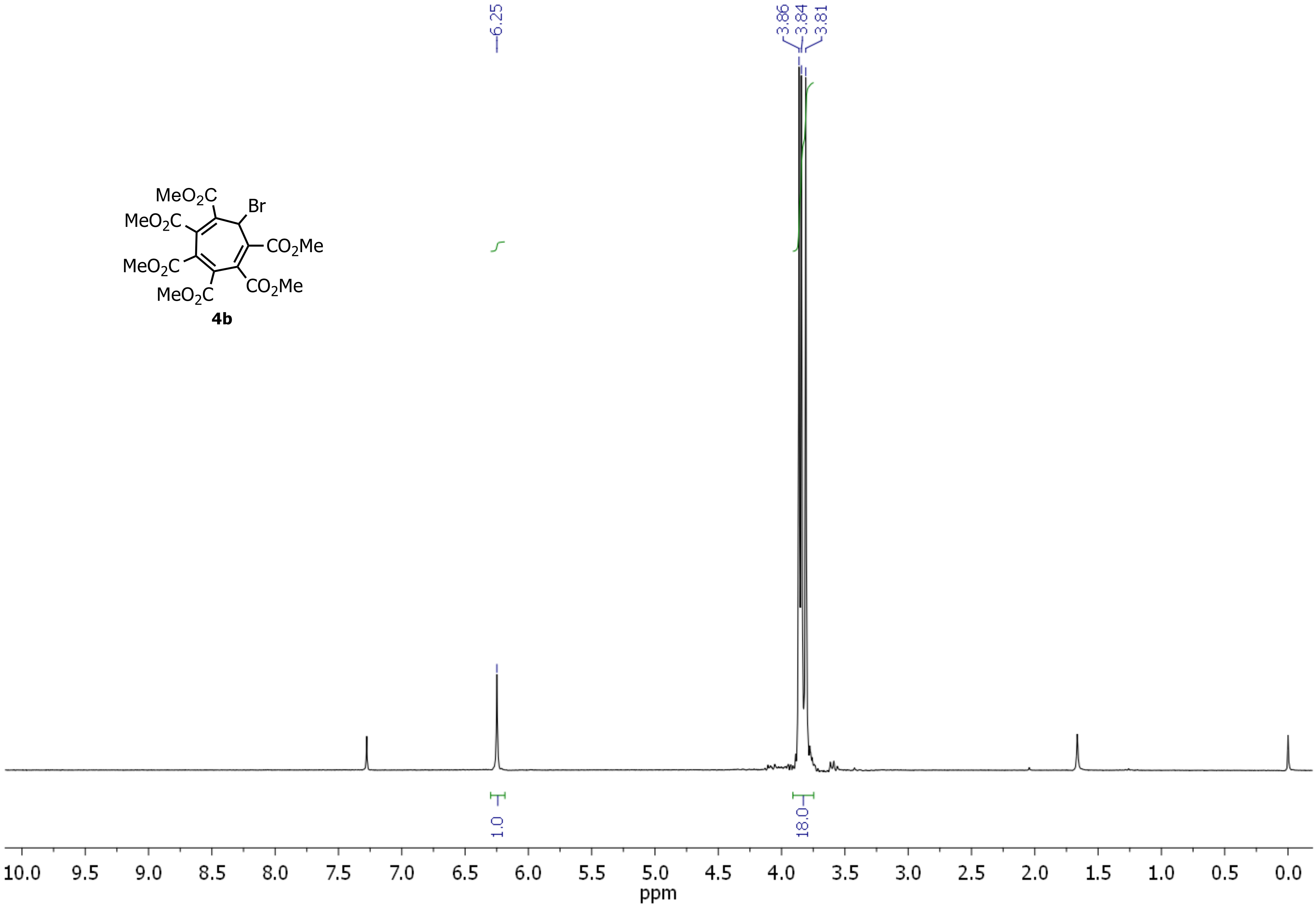
MeO2C
Br
MeO2C
CO2Me
MeO2C
CO2Me
MeO2C
6.25
3.86
3.84
3.81
1.0
18.0
10.0
9.5
9.0
8.5
8.0
7.5
7.0
6.5
6.0
5.5
5.0
4.5
4.0
3.5
3.0
2.5
2.0
1.5
1.0
0.5
0.0
ppm

4b

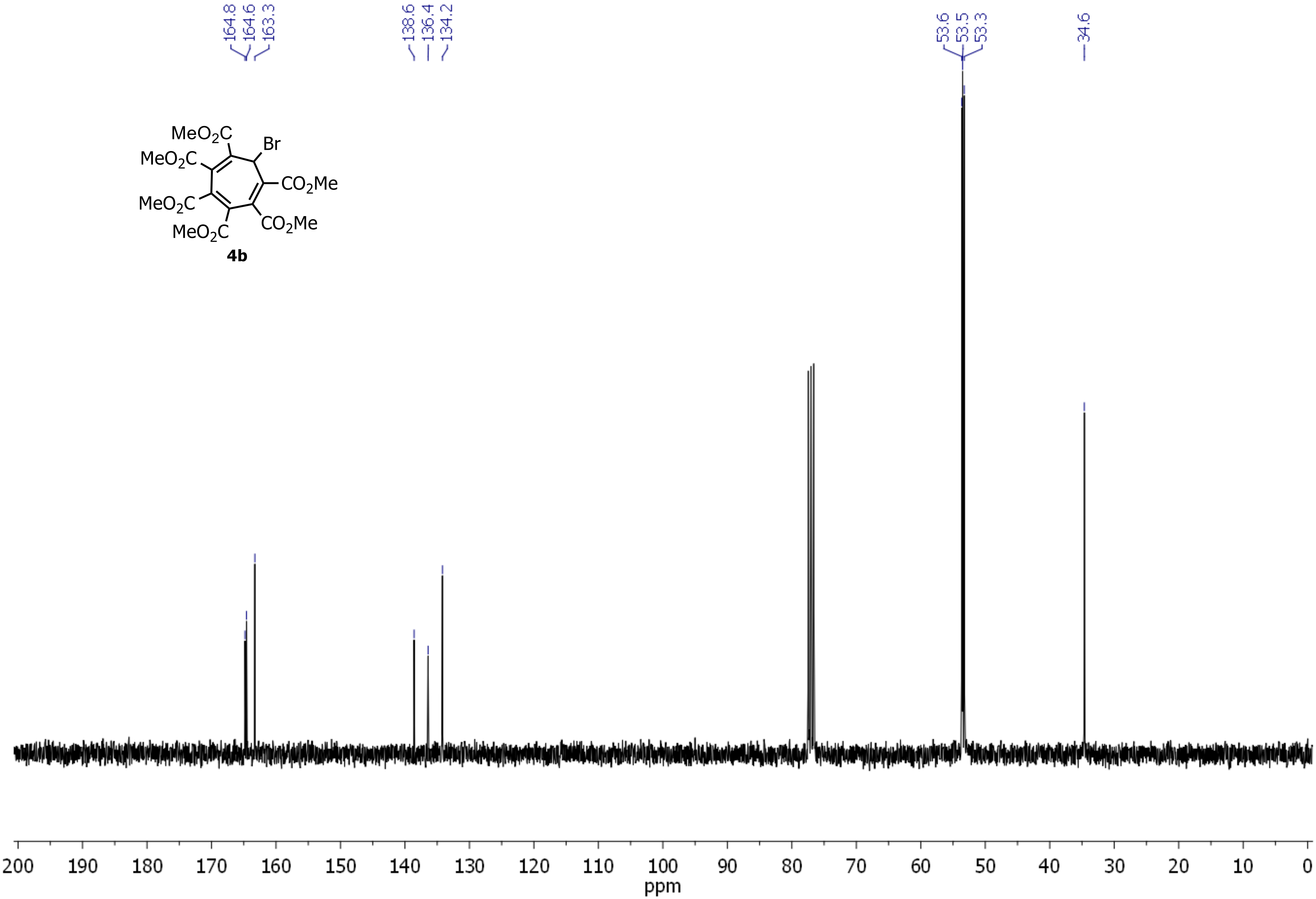
164.8
164.6
163.3
138.6
136.4
134.2
53.6
53.5
53.3
34.6
MeO2C
Br
MeO2C
CO2Me
MeO2C
CO2Me
MeO2C
4b
200 190 180 170 160 150 140 130 120 110 100 90 80 70 60 50 40 30 20 10 0
ppm

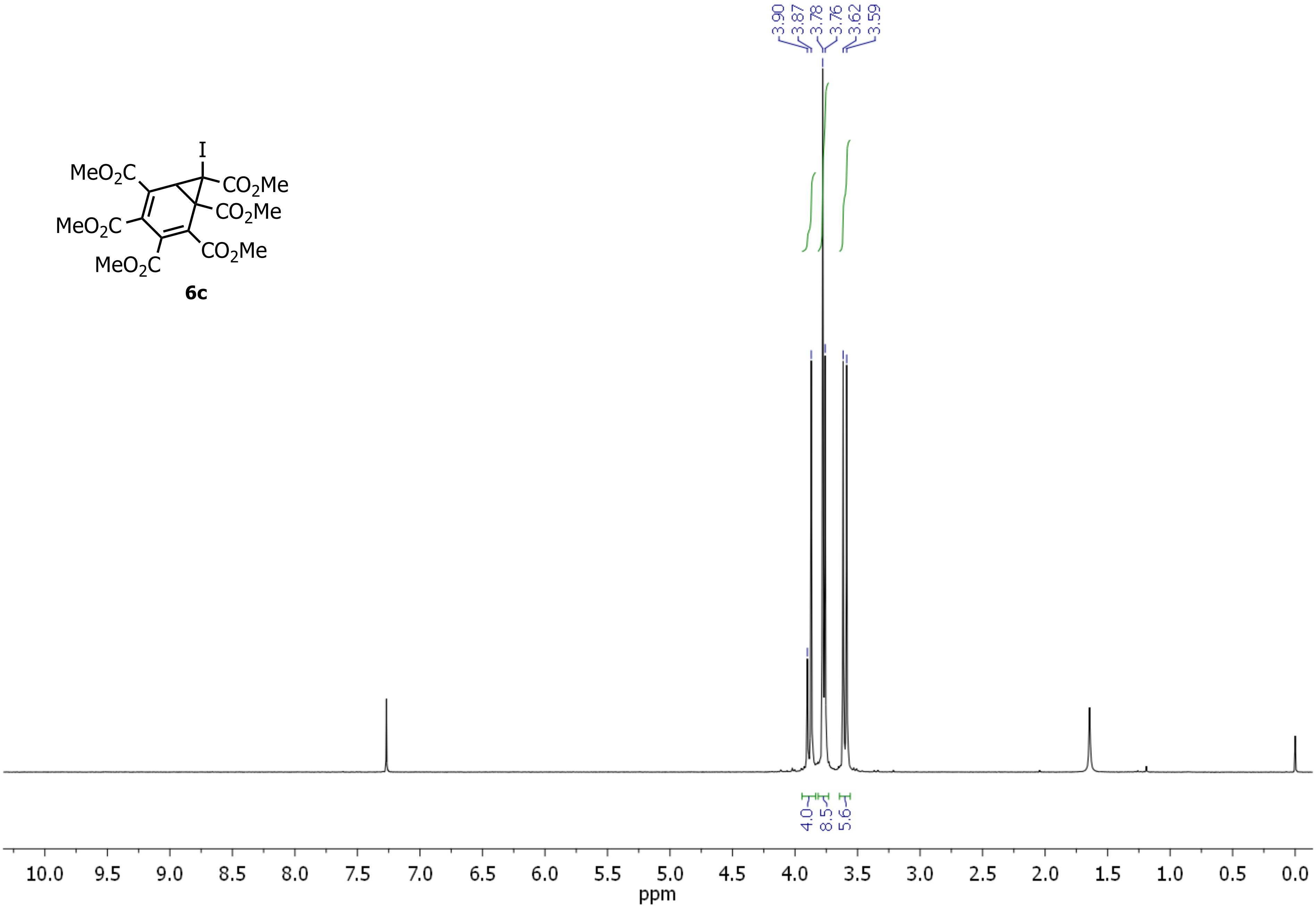
I
MeO2C
CO2Me
CO2Me
MeO2C
CO2Me
MeO2C
6c
3.90
3.87
3.78
3.76
3.62
3.59
4.0
8.5
5.6
10.0
9.5
9.0
8.5
8.0
7.5
7.0
6.5
6.0
5.5
5.0
4.5
4.0
3.5
3.0
2.5
2.0
1.5
1.0
0.5
0.0
ppm

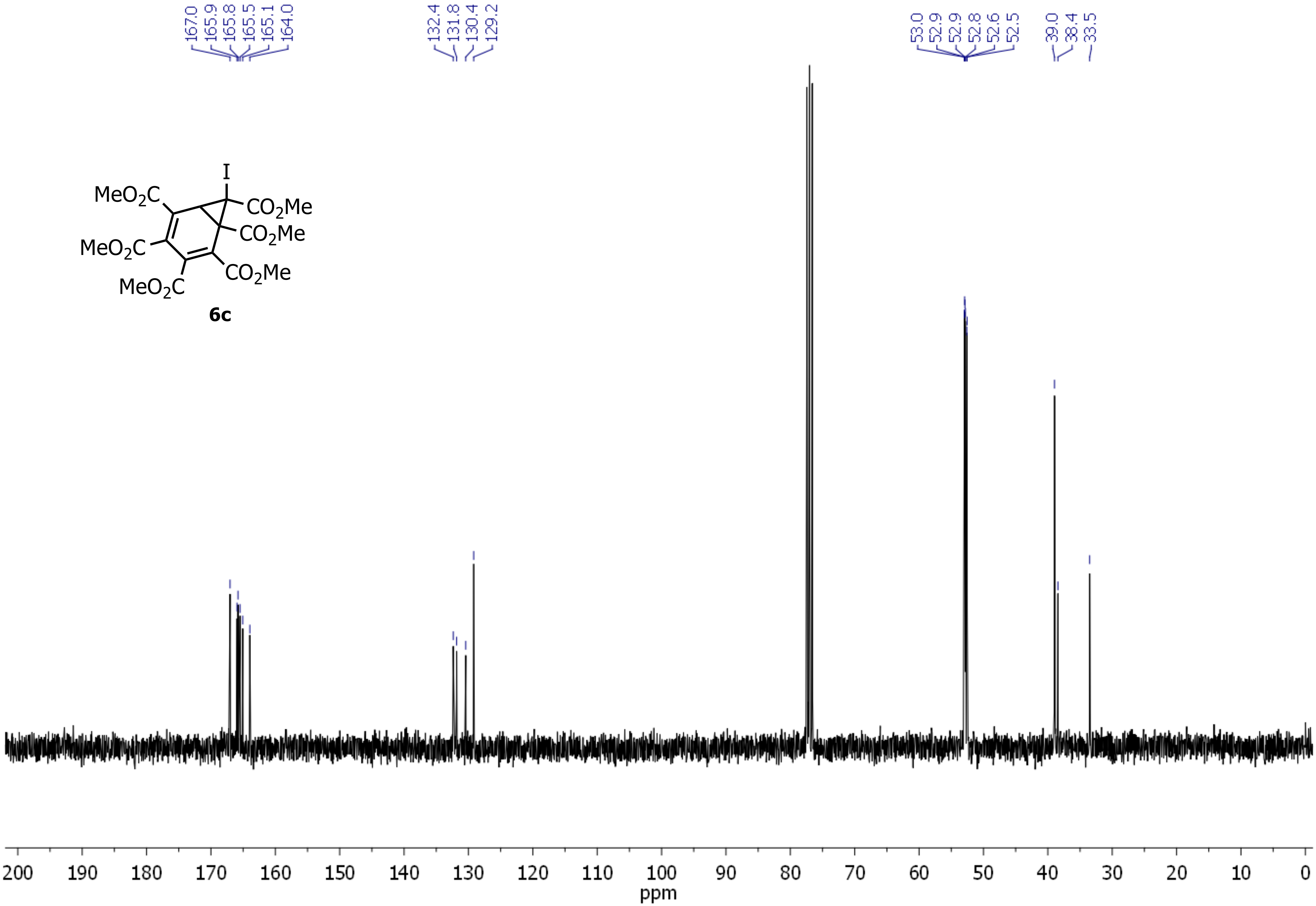
167.0
165.9
165.8
165.5
165.1
164.0
132.4
131.8
130.4
129.2
53.0
52.9
52.9
52.8
52.6
52.5
39.0
38.4
33.5
I
MeO2C
CO2Me
CO2Me
MeO2C
CO2Me
MeO2C
6c
200
190
180
170
160
150
140
130
120
110
100
90
80
70
60
50
40
30
20
10
0
ppm

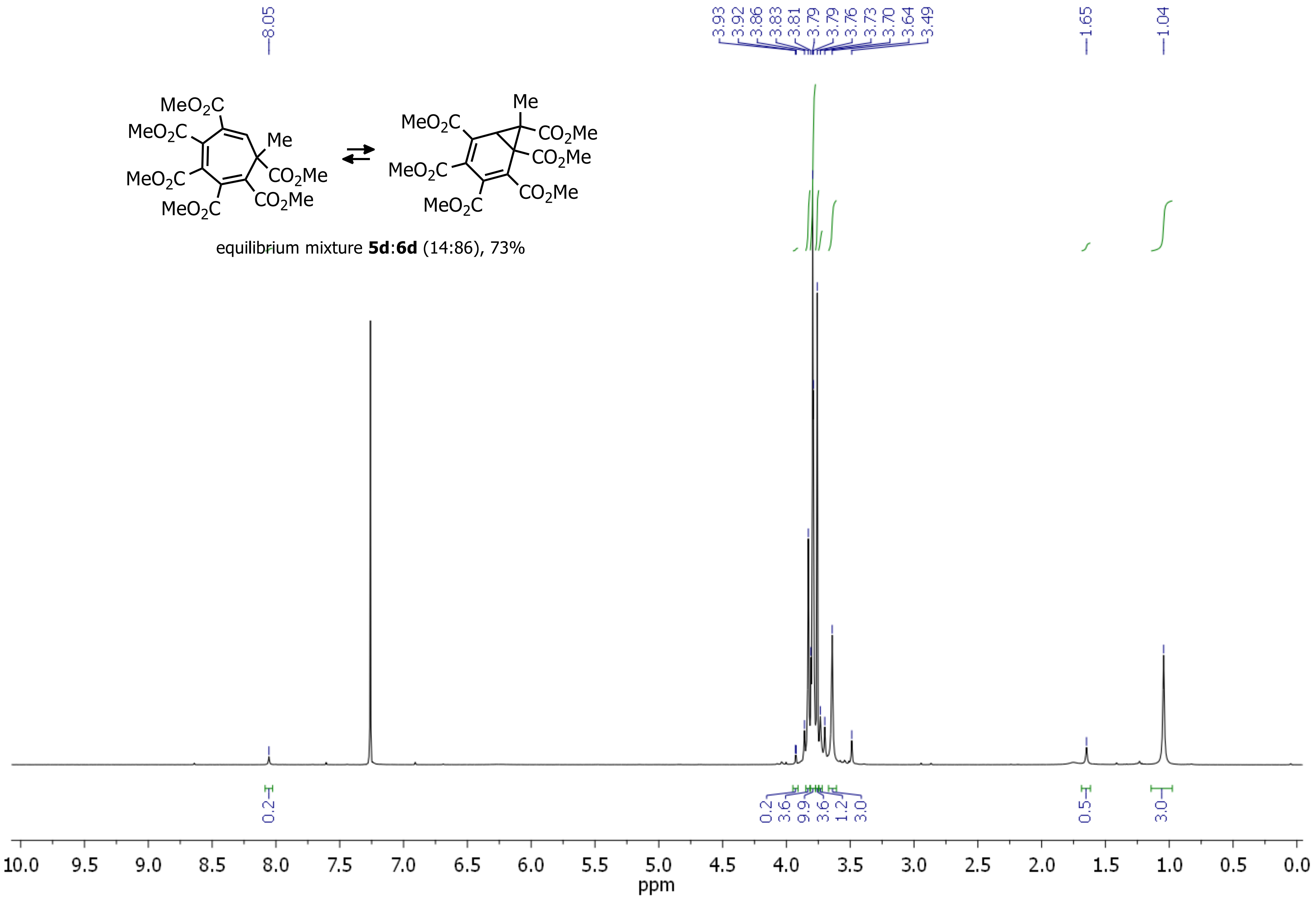
MeO2C
MeO2C
Me
CO2Me
MeO2C
MeO2C
CO2Me
Me
MeO2C
CO2Me
CO2Me
MeO2C
CO2Me
MeO2C
equilibrium mixture 5d:6d (14:86), 73%
8.05
3.93
3.92
3.86
3.83
3.81
3.79
3.79
3.76
3.73
3.70
3.64
3.49
1.65
1.04
0.2
0.2
3.6
9.9
3.6
1.2
3.0
0.5
3.0
10.0 9.5 9.0 8.5 8.0 7.5 7.0 6.5 6.0 5.5 5.0 4.5 4.0 3.5 3.0 2.5 2.0 1.5 1.0 0.5 0.0
ppm

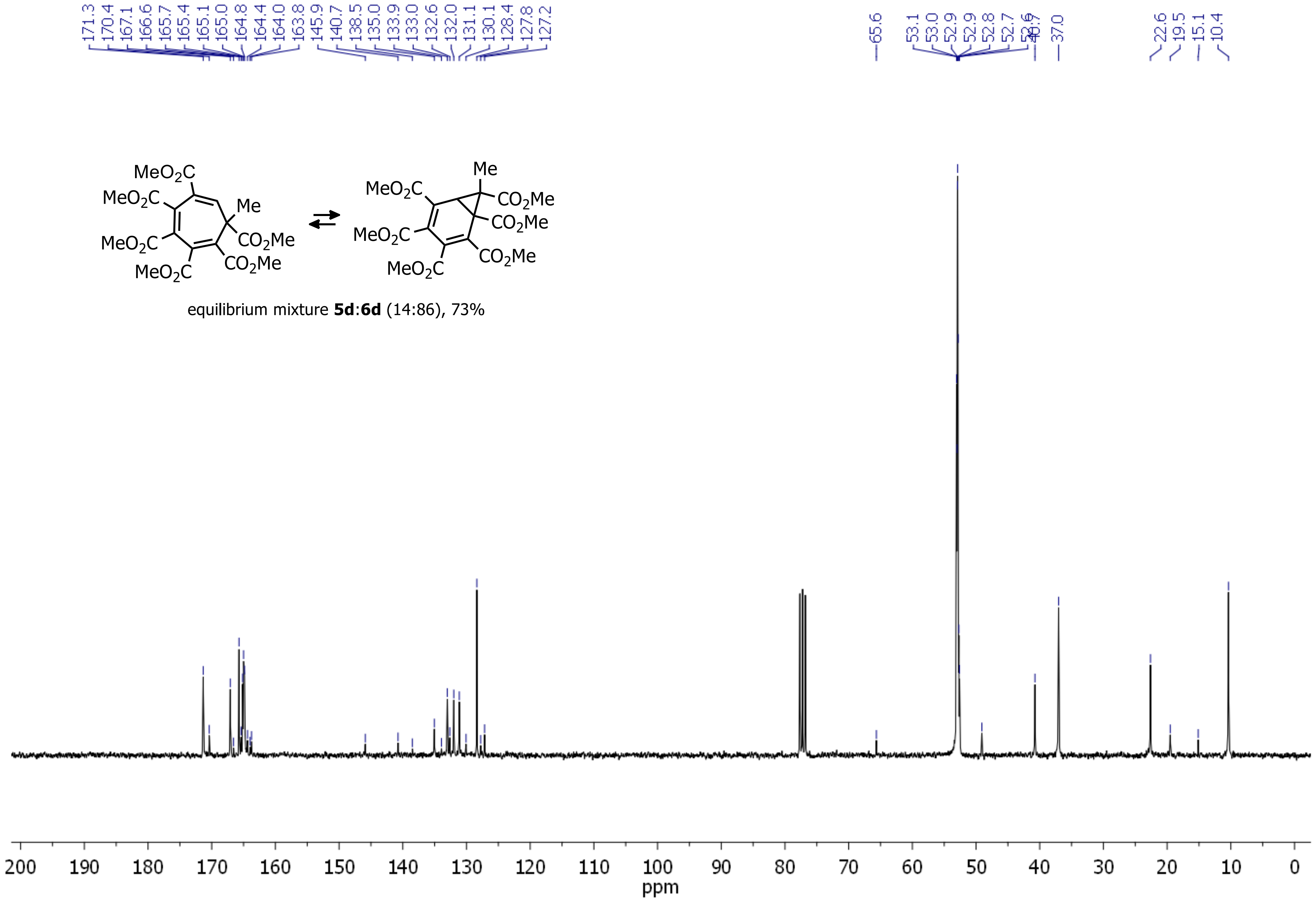

equilibrium mixture **5d**:**6d** (14:86), 73%

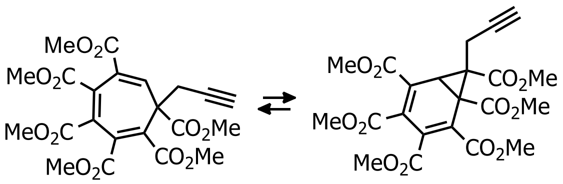

equilibrium mixture **5e**:**6e** (23:77), 96%

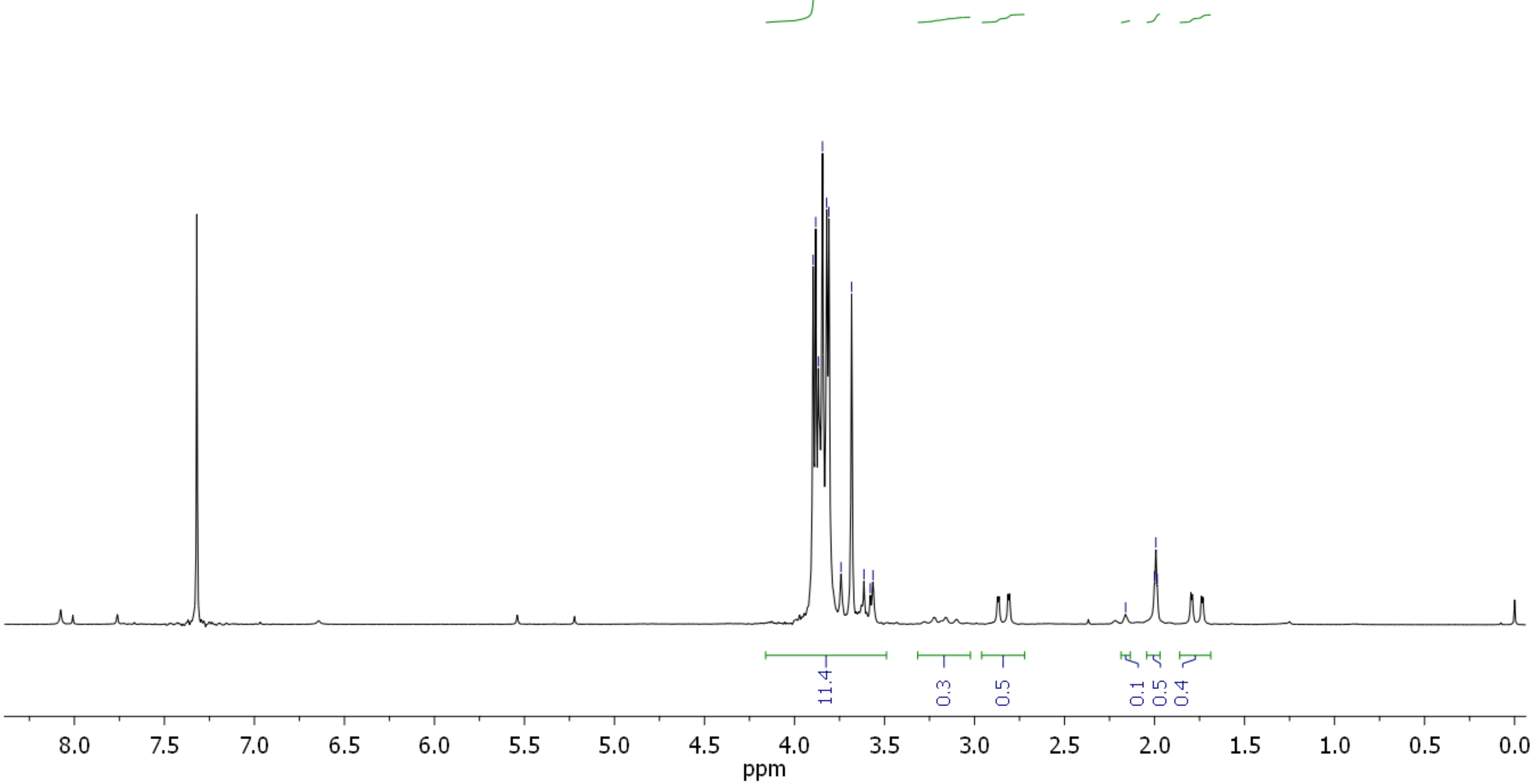

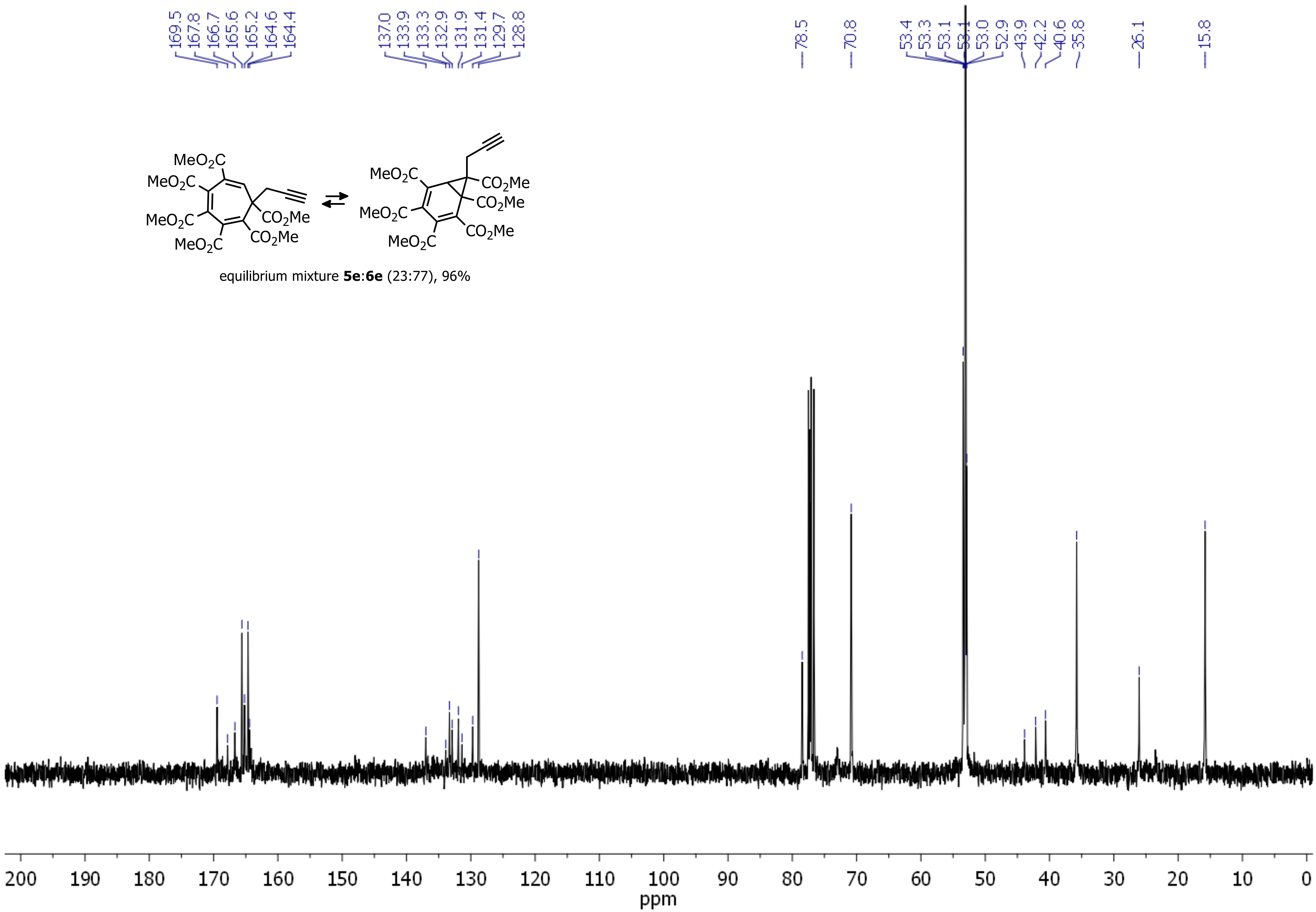

equilibrium mixture **5e**:**6e** (23:77), 96%

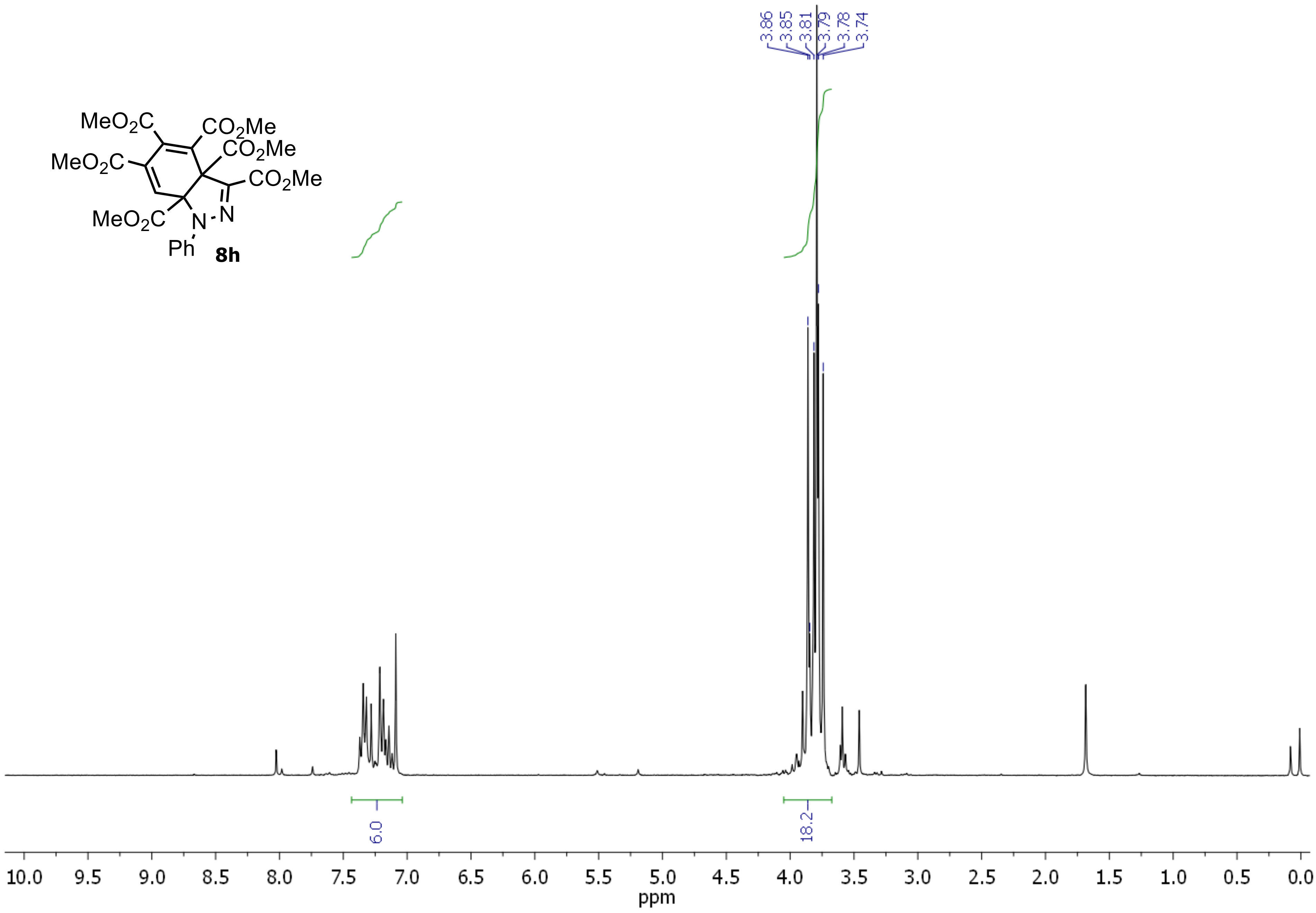
MeO2C
CO2Me
CO2Me
MeO2C
CO2Me
MeO2C
N
N
Ph
8h
3.86
3.85
3.81
3.79
3.78
3.74
6.0
18.2
10.0
9.5
9.0
8.5
8.0
7.5
7.0
6.5
6.0
5.5
5.0
4.5
4.0
3.5
3.0
2.5
2.0
1.5
1.0
0.5
0.0
ppm

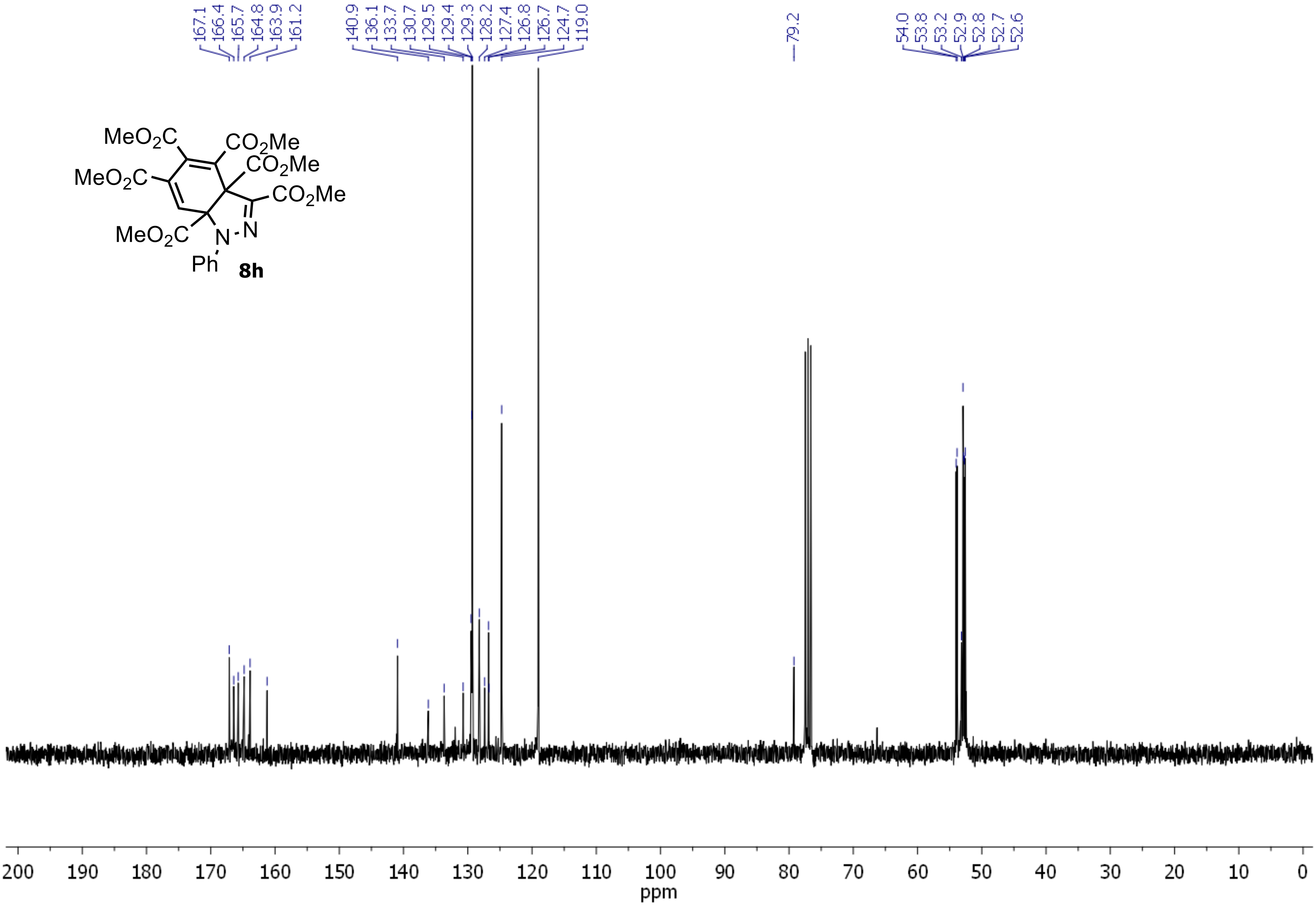

167.1
166.4
165.7
164.8
163.9
161.2
140.9
136.1
133.7
130.7
129.5
129.4
129.3
128.2
127.4
126.8
126.7
124.7
119.0
79.2
54.0
53.8
53.2
52.9
52.8
52.7
52.6
MeO2C
CO2Me
CO2Me
MeO2C
CO2Me
MeO2C
N
N
Ph
8h
200 190 180 170 160 150 140 130 120 110 100 90 80 70 60 50 40 30 20 10 0
ppm

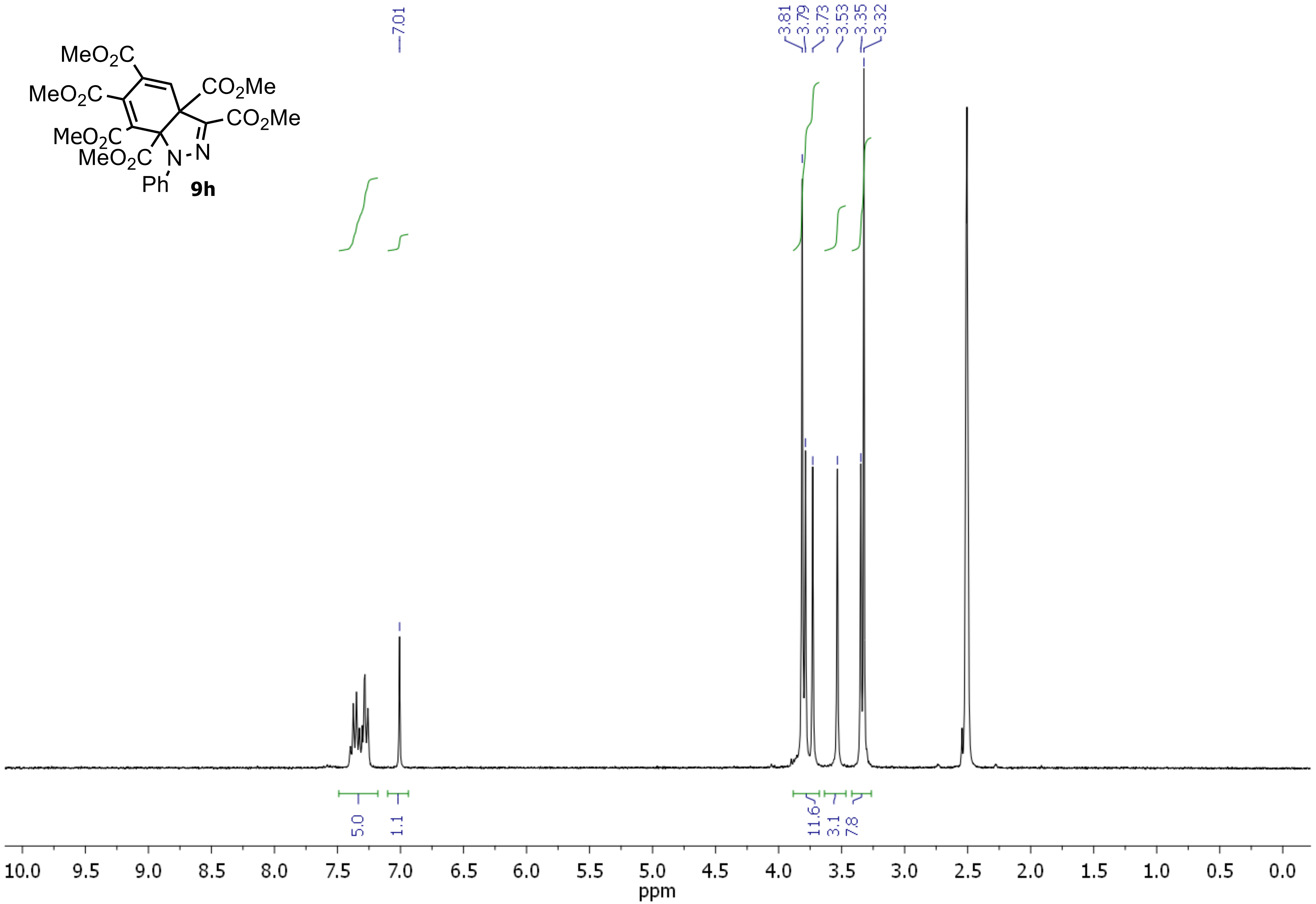
MeO2C
MeO2C
MeO2C
MeO2C
CO2Me
CO2Me
N
N
Ph
9h
7.01
3.81
3.79
3.73
3.53
3.35
3.32
5.0
1.1
11.6
3.1
7.8
10.0
9.5
9.0
8.5
8.0
7.5
7.0
6.5
6.0
5.5
5.0
4.5
4.0
3.5
3.0
2.5
2.0
1.5
1.0
0.5
0.0
ppm

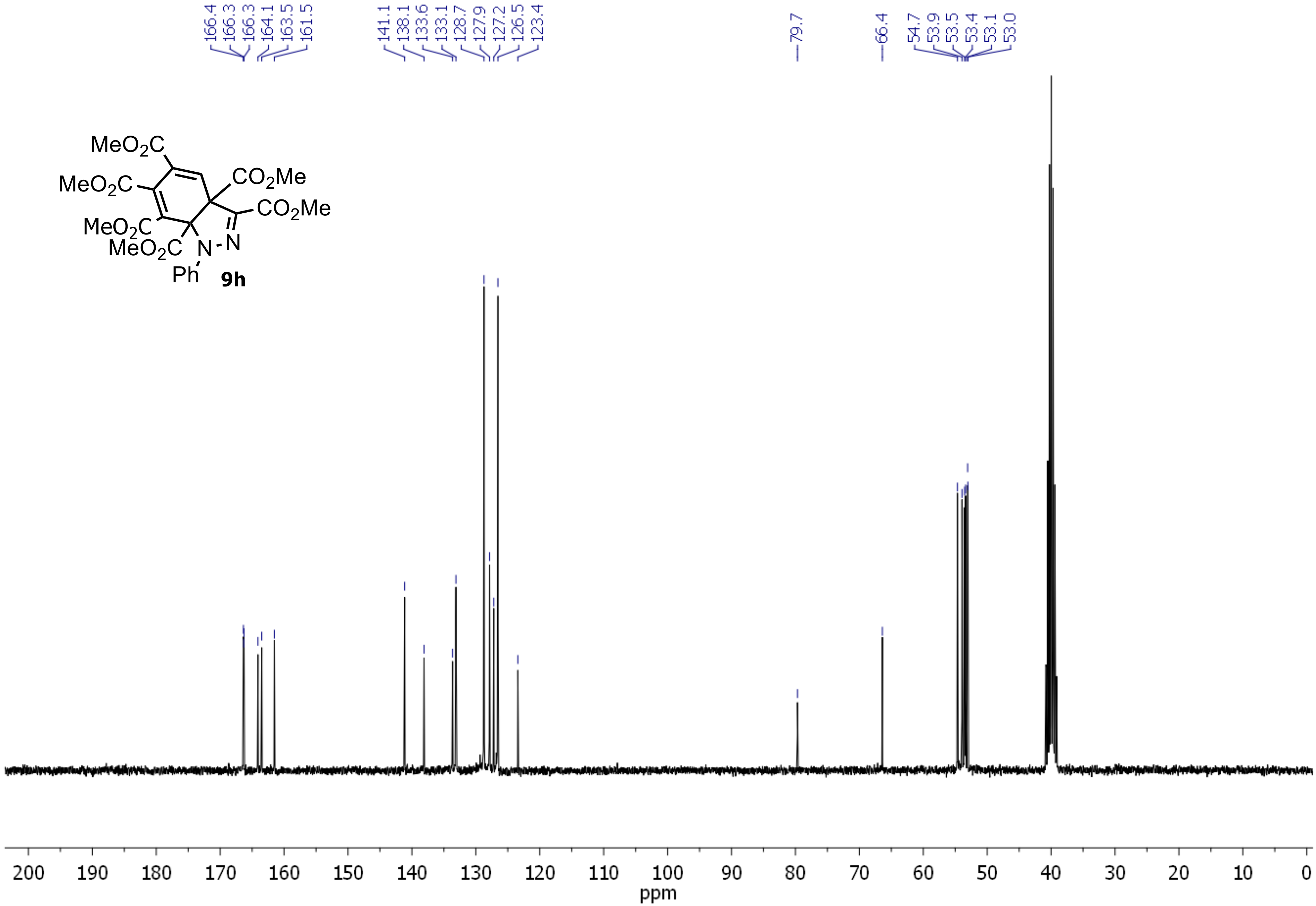

166.4
166.3
166.3
164.1
163.5
161.5
141.1
138.1
133.6
133.1
128.7
127.9
127.2
126.5
123.4
79.7
66.4
54.7
53.9
53.5
53.4
53.1
53.0
MeO2C
MeO2C
CO2Me
CO2Me
MeO2C
MeO2C
N
N
Ph
9h
200
190
180
170
160
150
140
130
120
110
100
90
80
70
60
50
40
30
20
10
0
ppm

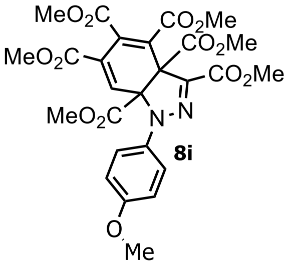
MeO2C
CO2Me
CO2Me
MeO2C
CO2Me
MeO2C
N
N
O
Me

8i

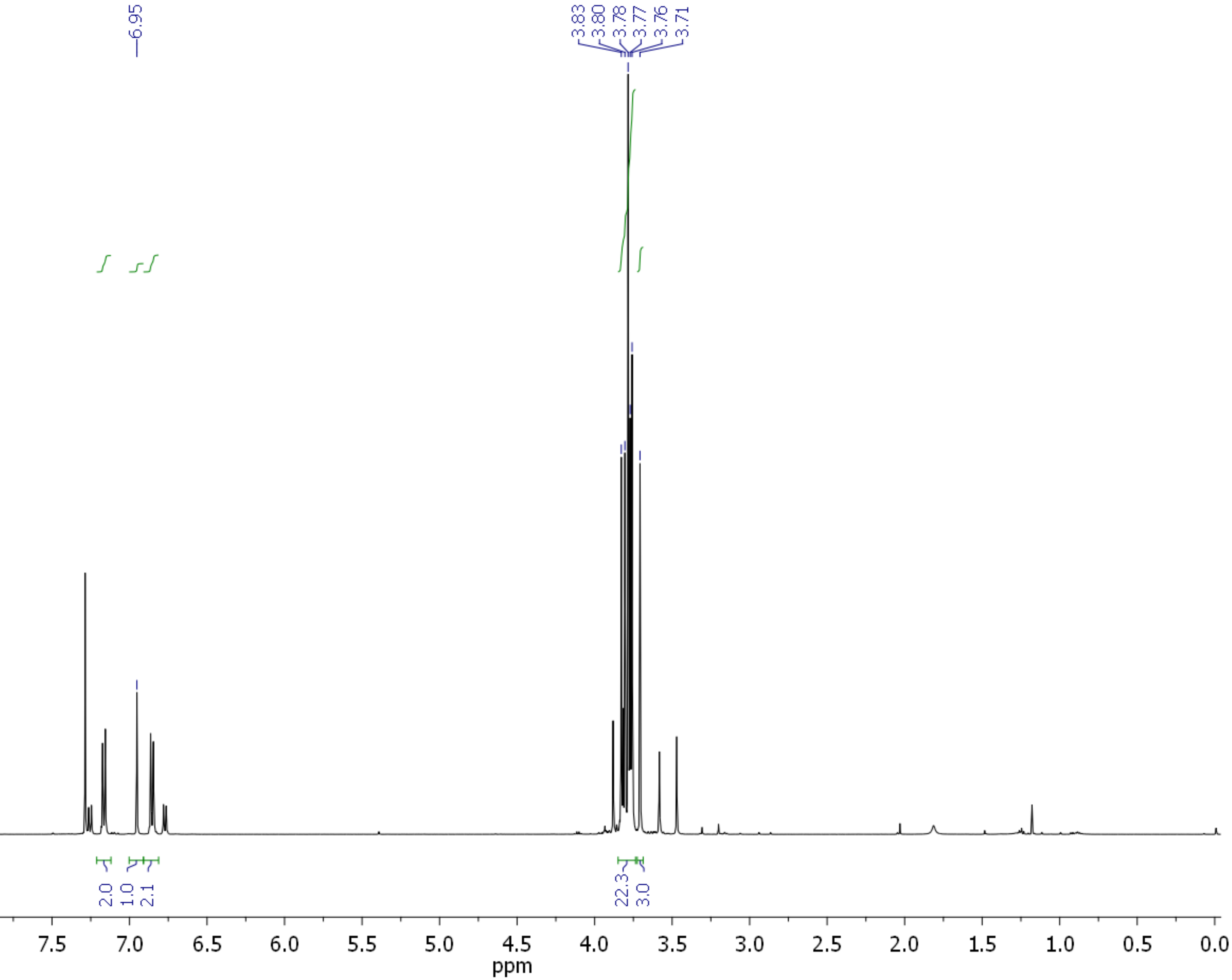
6.95
3.83
3.80
3.78
3.77
3.76
3.71
2.0
1.0
2.1
22.3
3.0
9.0
8.5
8.0
7.5
7.0
6.5
6.0
5.5
5.0
4.5
4.0
3.5
3.0
2.5
2.0
1.5
1.0
0.5
0.0
ppm

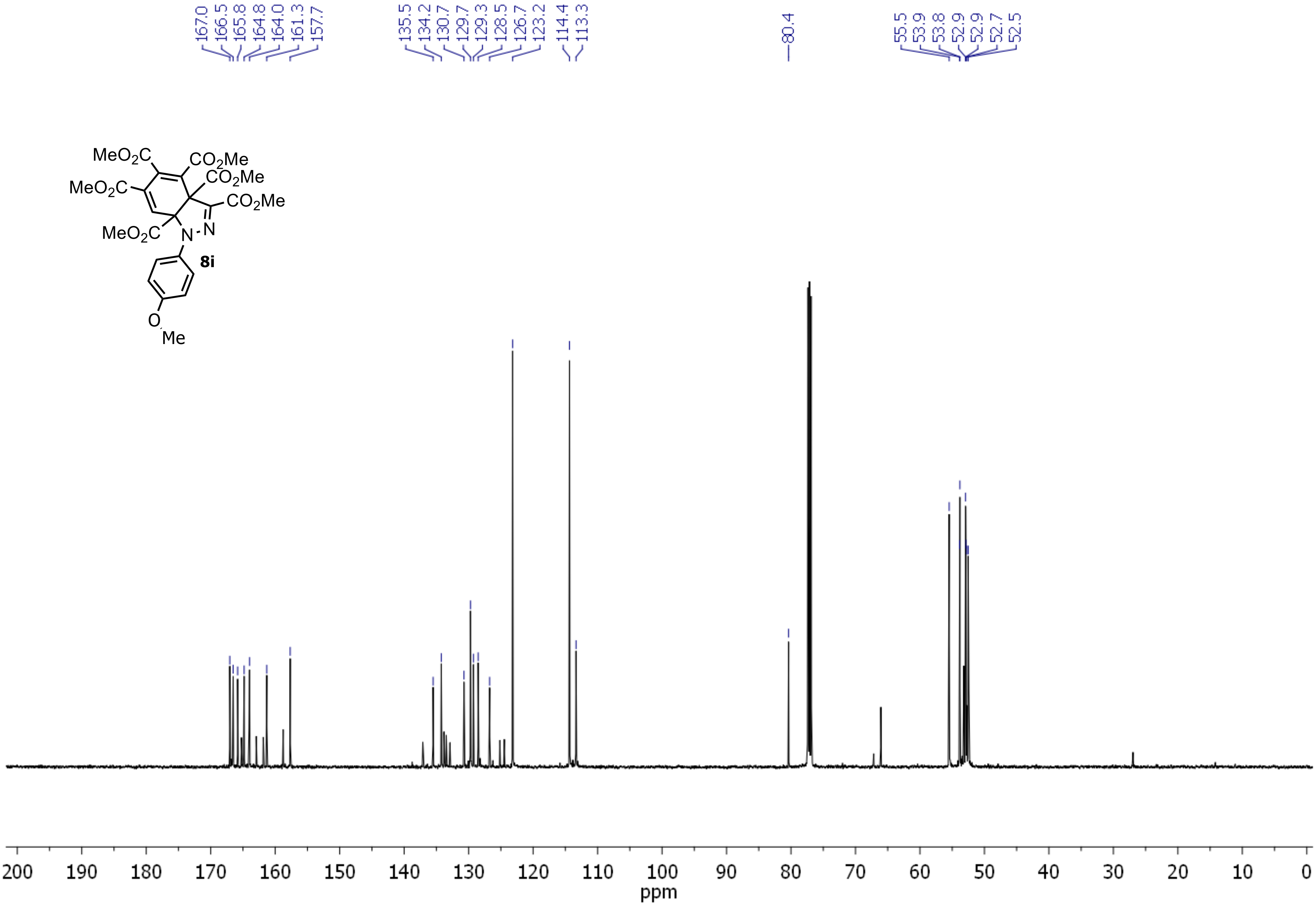
167.0
166.5
165.8
164.8
164.0
161.3
157.7
135.5
134.2
130.7
129.7
129.3
128.5
126.7
123.2
114.4
113.3
80.4
55.5
53.9
53.8
52.9
52.9
52.7
52.5
MeO2C
CO2Me
CO2Me
MeO2C
CO2Me
MeO2C
N
N
8i
O
Me
200 190 180 170 160 150 140 130 120 110 100 90 80 70 60 50 40 30 20 10 0
ppm

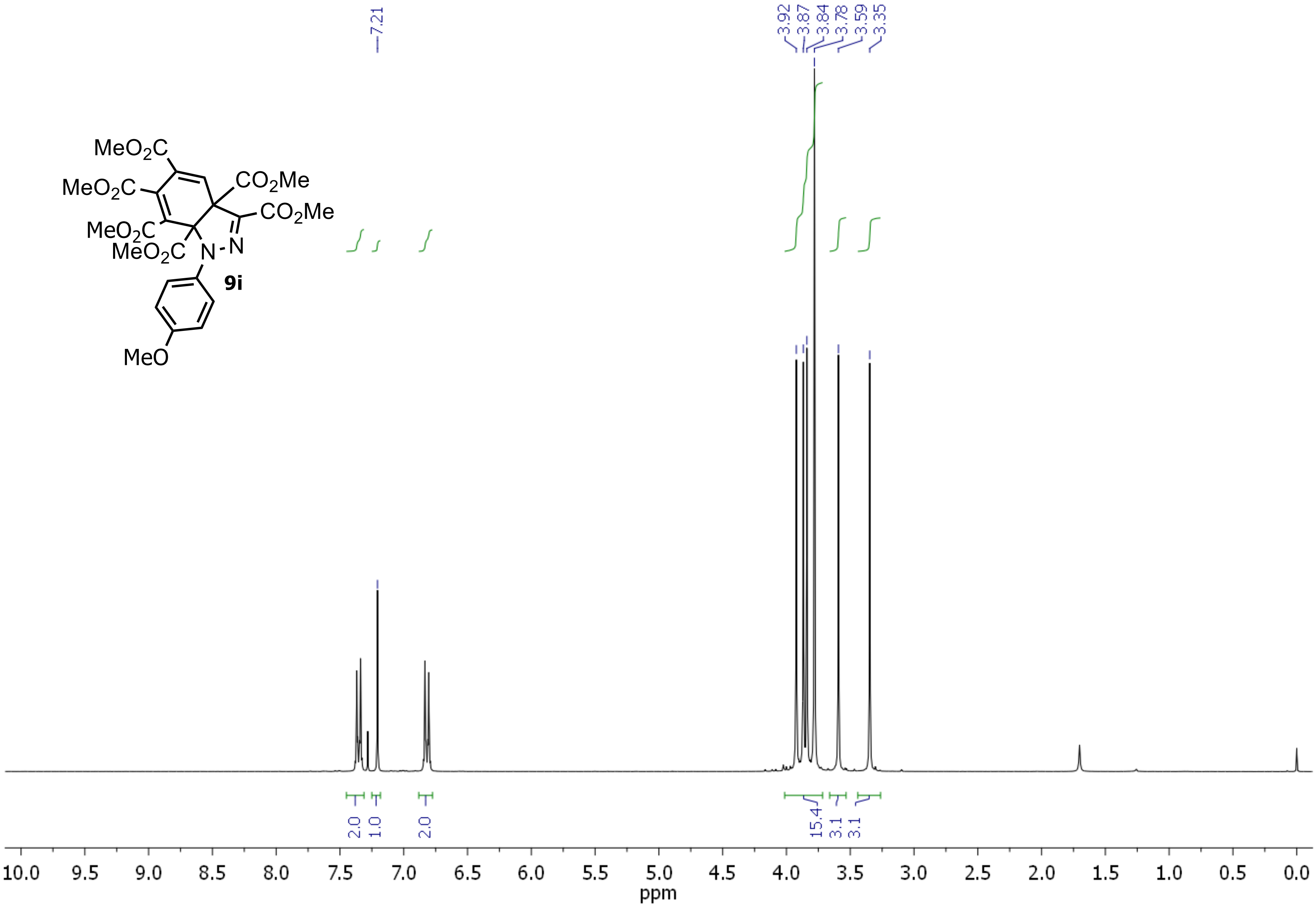
MeO2C
MeO2C
MeO2C
MeO2C
CO2Me
CO2Me
N
N
MeO
9i
7.21
3.92
3.87
3.84
3.78
3.59
3.35
2.0
1.0
2.0
15.4
3.1
3.1
10.0 9.5 9.0 8.5 8.0 7.5 7.0 6.5 6.0 5.5 5.0 4.5 4.0 3.5 3.0 2.5 2.0 1.5 1.0 0.5 0.0
ppm

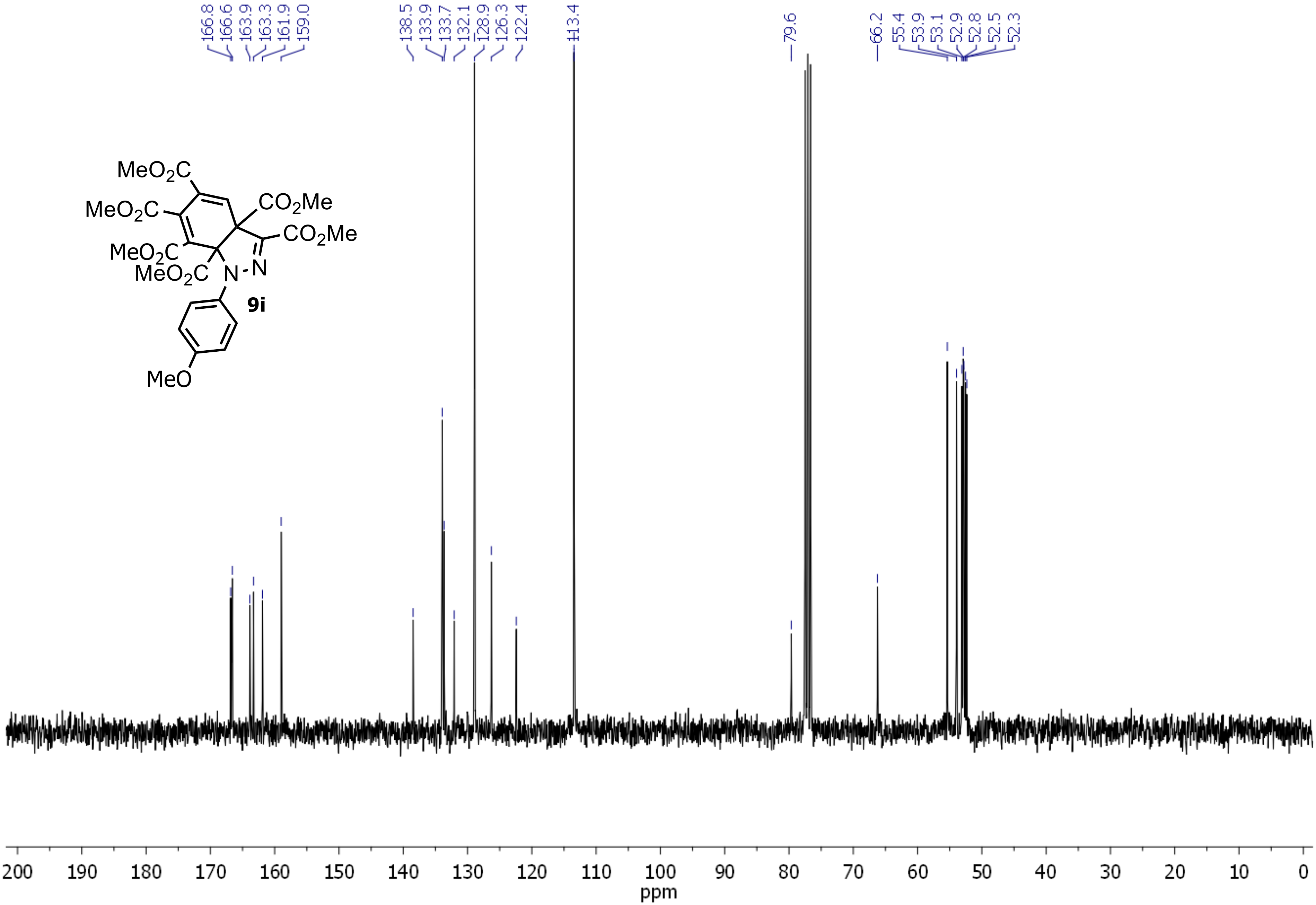

166.8
166.6
163.9
163.3
161.9
159.0
138.5
133.9
133.7
132.1
128.9
126.3
122.4
113.4
79.6
66.2
55.4
53.9
53.1
52.9
52.8
52.5
52.3
MeO2C
MeO2C
CO2Me
CO2Me
MeO2C
MeO2C
N
N
9i
MeO
200 190 180 170 160 150 140 130 120 110 100 90 80 70 60 50 40 30 20 10 0
ppm

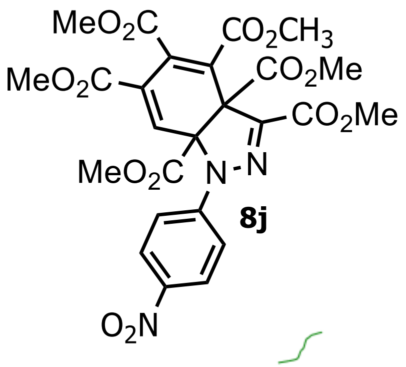
MeO2C
CO2CH3
CO2Me
MeO2C
CO2Me
MeO2C
N
N
O2N

8j

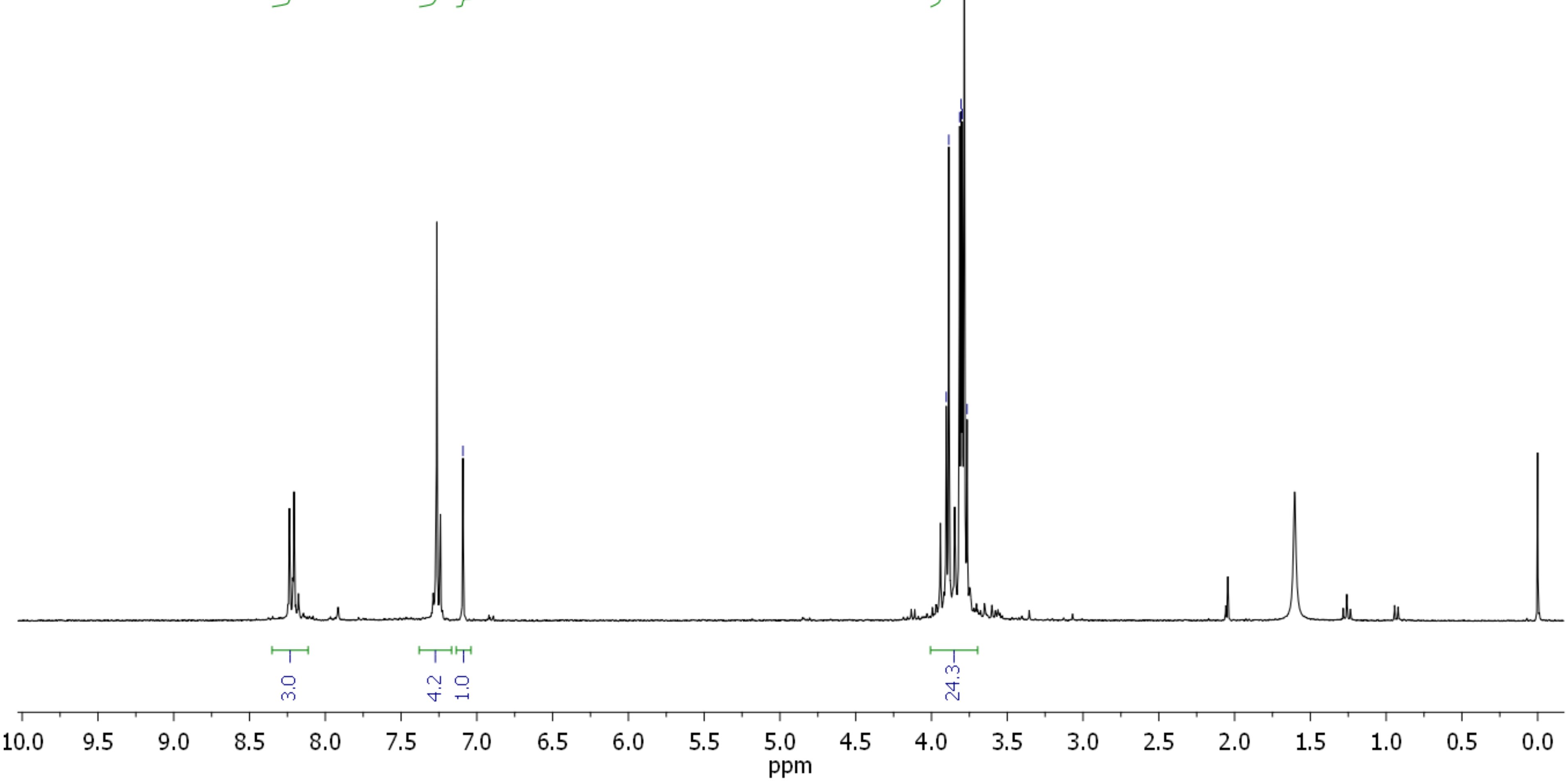
7.09
3.90
3.89
3.81
3.81
3.80
3.78
3.77
3.0
4.2
1.0
24.3
10.0
9.5
9.0
8.5
8.0
7.5
7.0
6.5
6.0
5.5
5.0
4.5
4.0
3.5
3.0
2.5
2.0
1.5
1.0
0.5
0.0
ppm

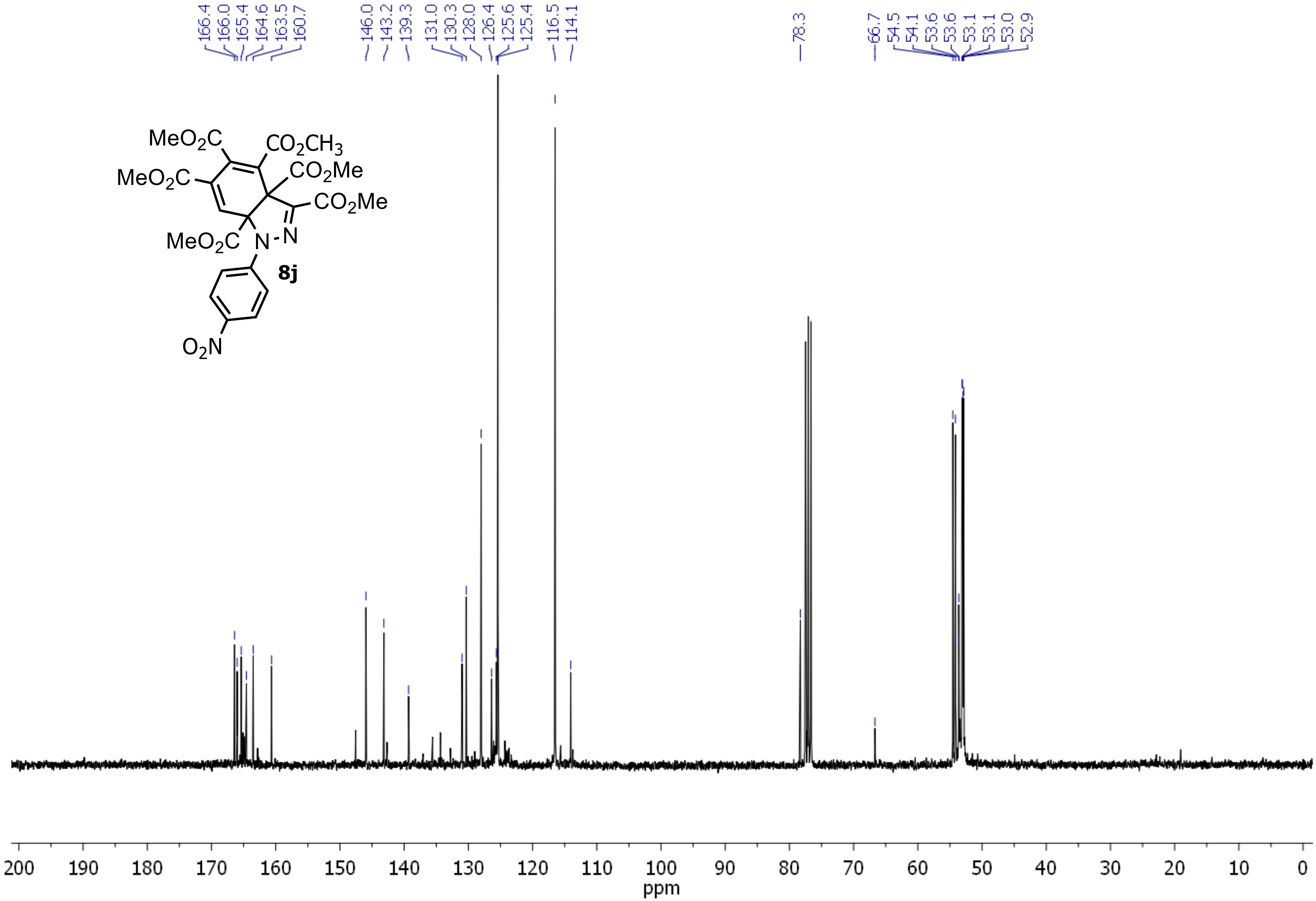
166.4
166.0
165.4
164.6
163.5
160.7
146.0
143.2
139.3
131.0
130.3
128.0
126.4
125.6
125.4
116.5
114.1
78.3
66.7
54.5
54.1
53.6
53.6
53.1
53.1
53.0
52.9
MeO2C
CO2CH3
CO2Me
MeO2C
CO2Me
MeO2C
N
N
O2N
8j
200 190 180 170 160 150 140 130 120 110 100 90 80 70 60 50 40 30 20 10 0
ppm

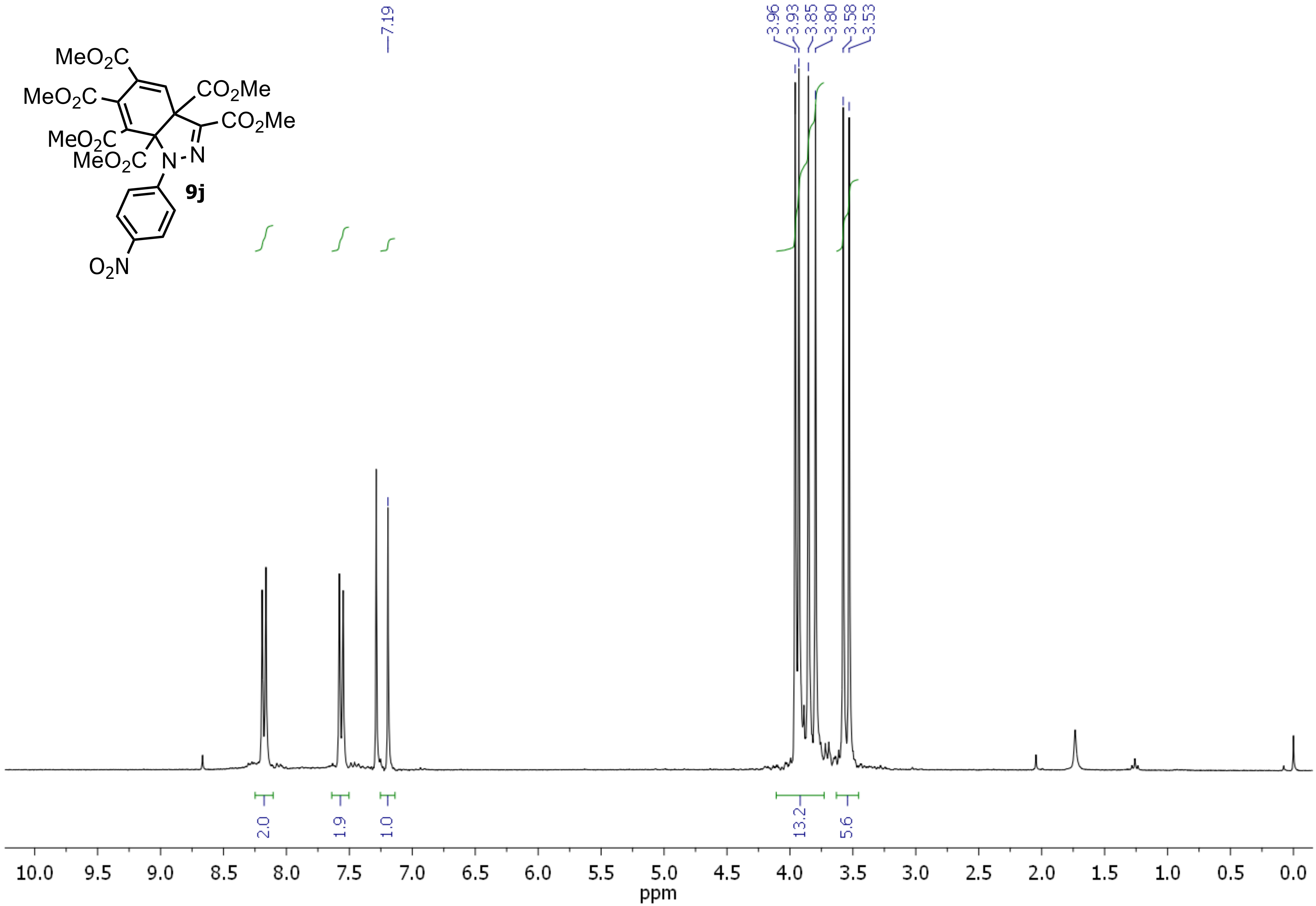
9j
7.19
3.96
3.93
3.85
3.80
3.58
3.53
2.0
1.9
1.0
13.2
5.6
ppm

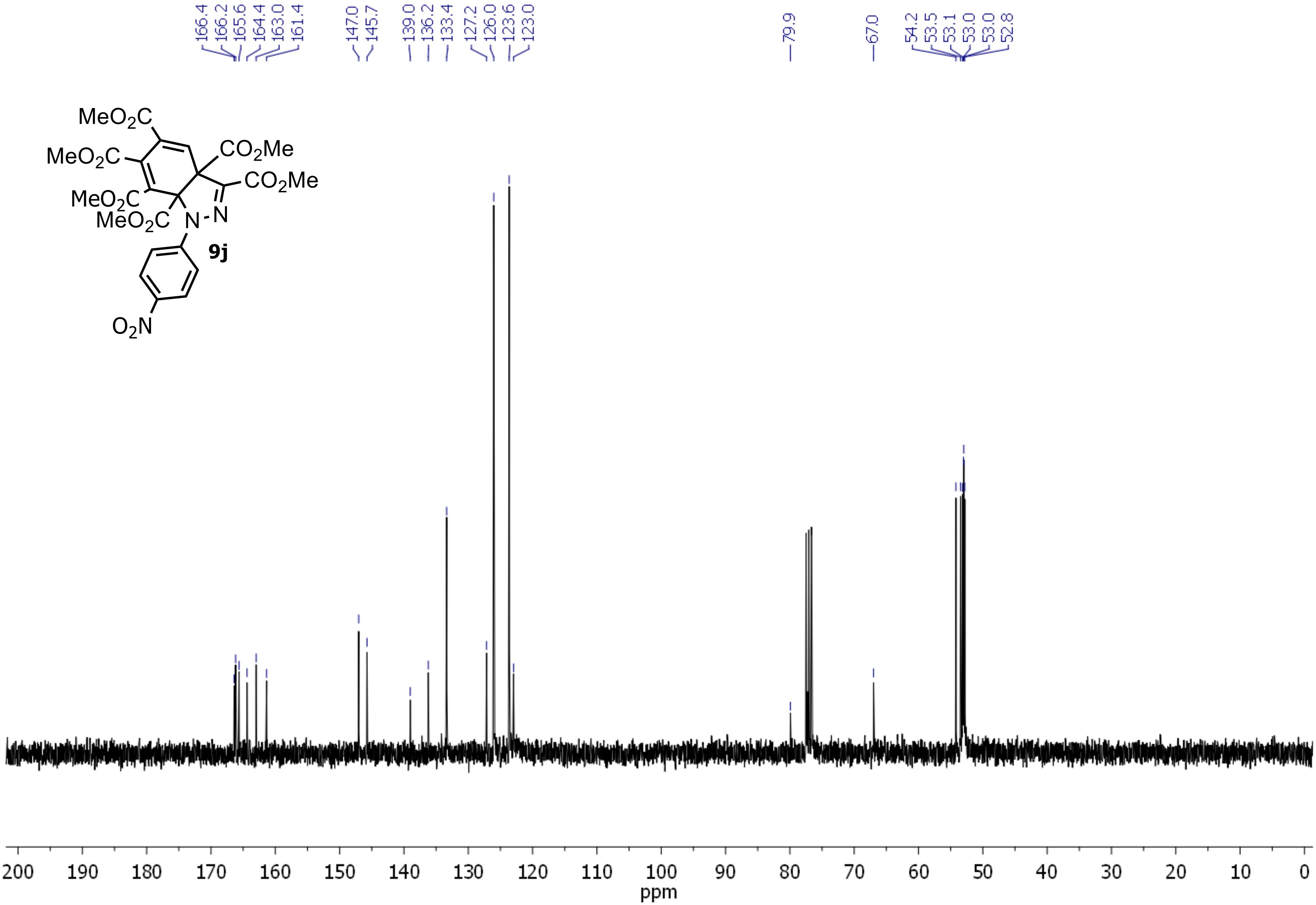
166.4
166.2
165.6
164.4
163.0
161.4
147.0
145.7
139.0
136.2
133.4
127.2
126.0
123.6
123.0
79.9
67.0
54.2
53.5
53.1
53.0
53.0
52.8
MeO2C
MeO2C
MeO2C
MeO2C
CO2Me
CO2Me
N
N
O2N
9j
200 190 180 170 160 150 140 130 120 110 100 90 80 70 60 50 40 30 20 10 0
ppm

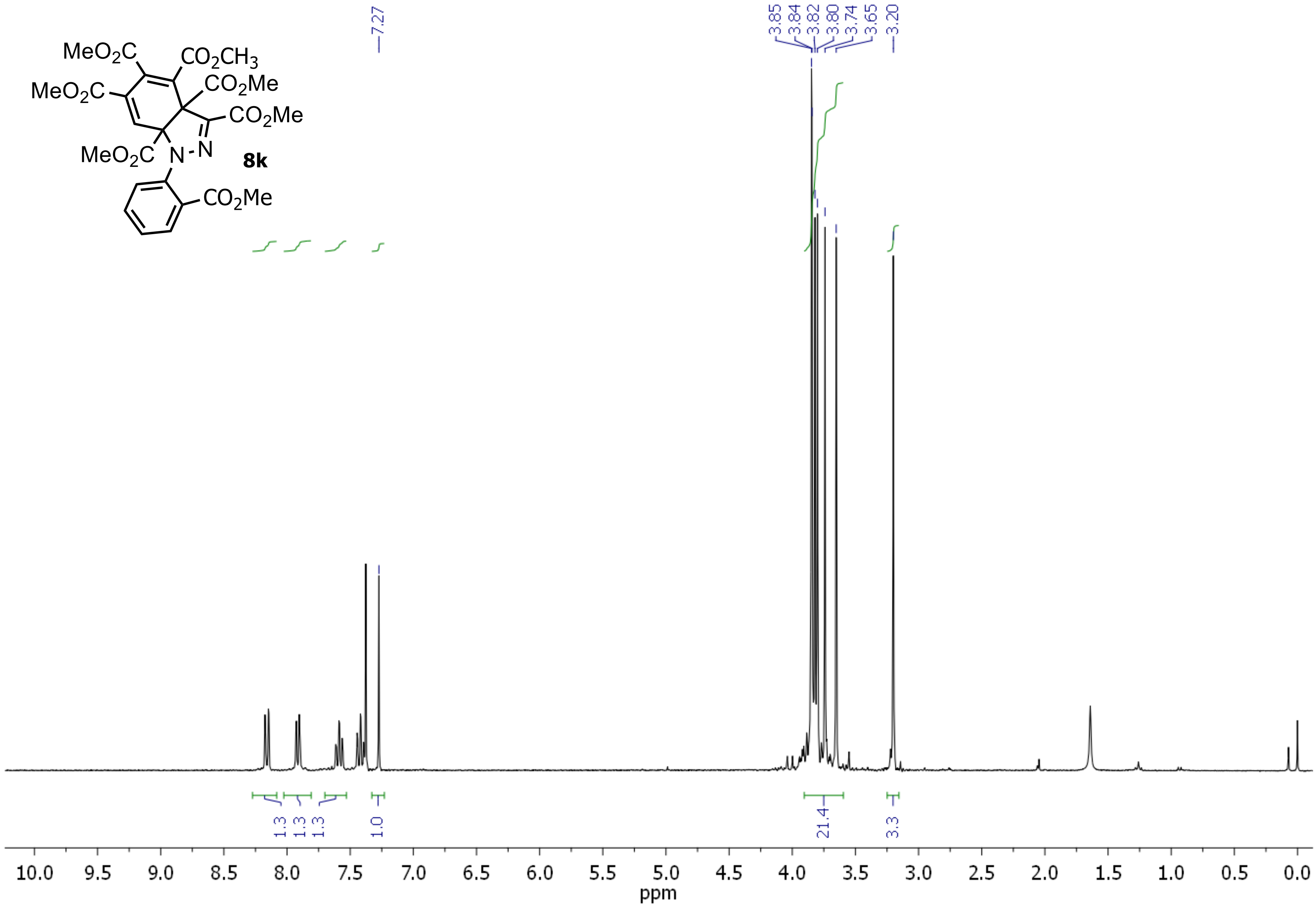
MeO2C
CO2CH3
CO2Me
MeO2C
CO2Me
MeO2C
N
N
CO2Me
8k
7.27
3.85
3.84
3.82
3.80
3.74
3.65
3.20
1.3
1.3
1.3
1.0
21.4
3.3
10.0
9.5
9.0
8.5
8.0
7.5
7.0
6.5
6.0
5.5
5.0
4.5
4.0
3.5
3.0
2.5
2.0
1.5
1.0
0.5
0.0
ppm

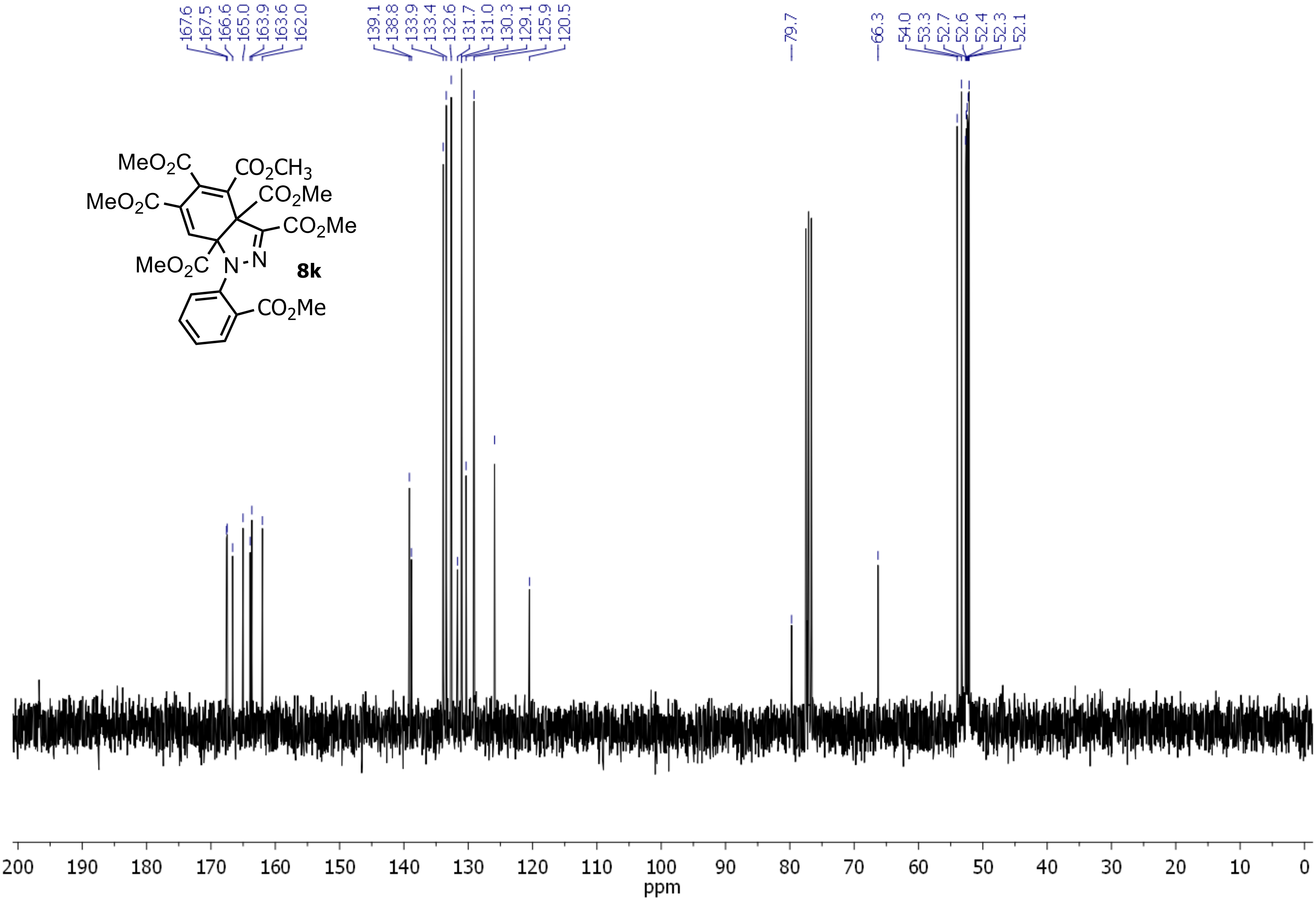
167.6
167.5
166.6
165.0
163.9
163.6
162.0
139.1
138.8
133.9
133.4
132.6
131.7
131.0
130.3
129.1
125.9
120.5
79.7
66.3
54.0
53.3
52.7
52.6
52.4
52.3
52.1
MeO_2C
CO_2CH_3
CO_2Me
MeO_2C
CO_2Me
MeO_2C
N
N
8k
CO_2Me
200 190 180 170 160 150 140 130 120 110 100 90 80 70 60 50 40 30 20 10 0
ppm

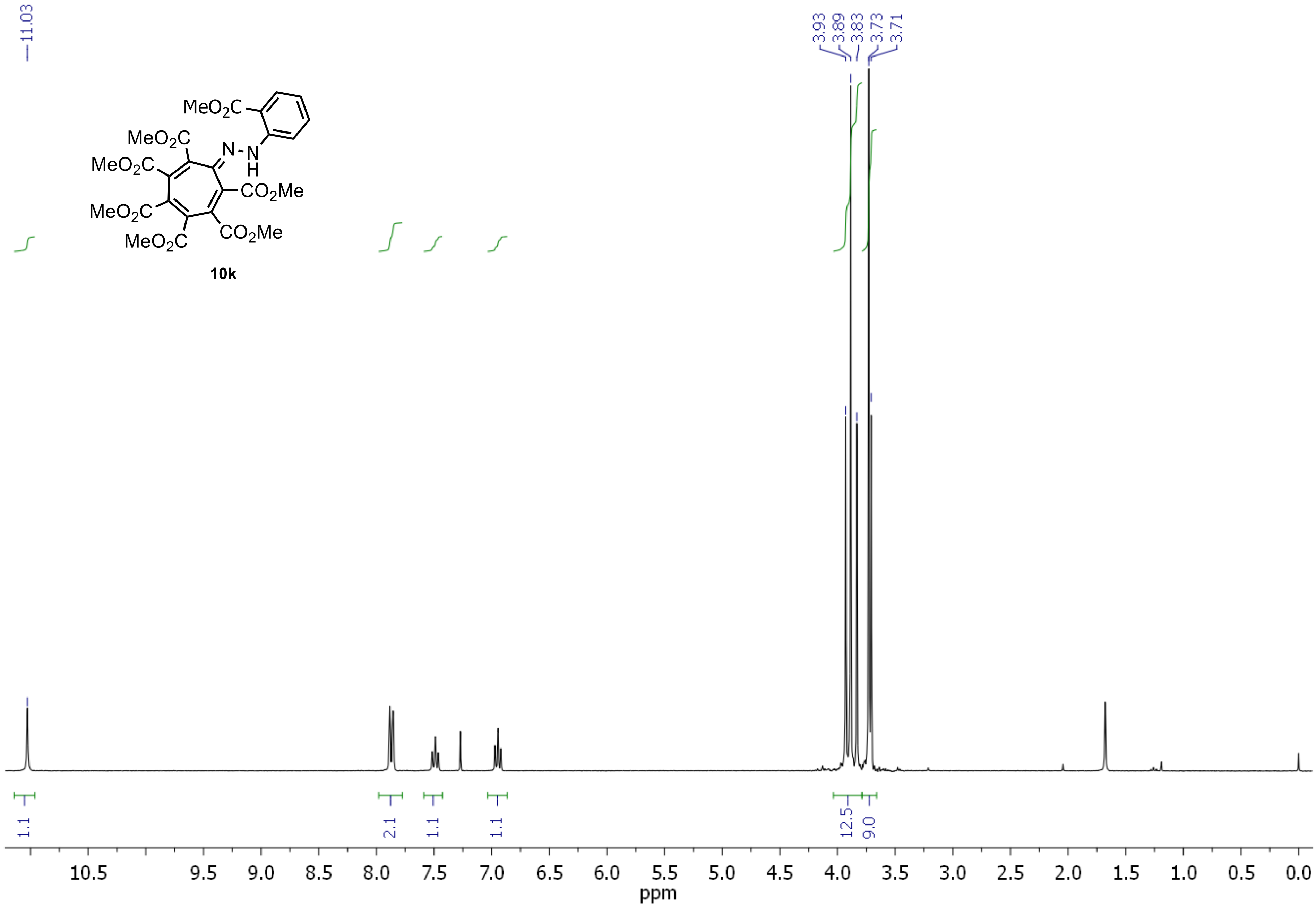

11.03
3.93
3.89
3.83
3.73
3.71
MeO2C
CO2Me
N-N
H
10k
1.1
2.1
1.1
1.1
12.5
9.0
10.5
9.5
9.0
8.5
8.0
7.5
7.0
6.5
6.0
5.5
5.0
4.5
4.0
3.5
3.0
2.5
2.0
1.5
1.0
0.5
0.0
ppm

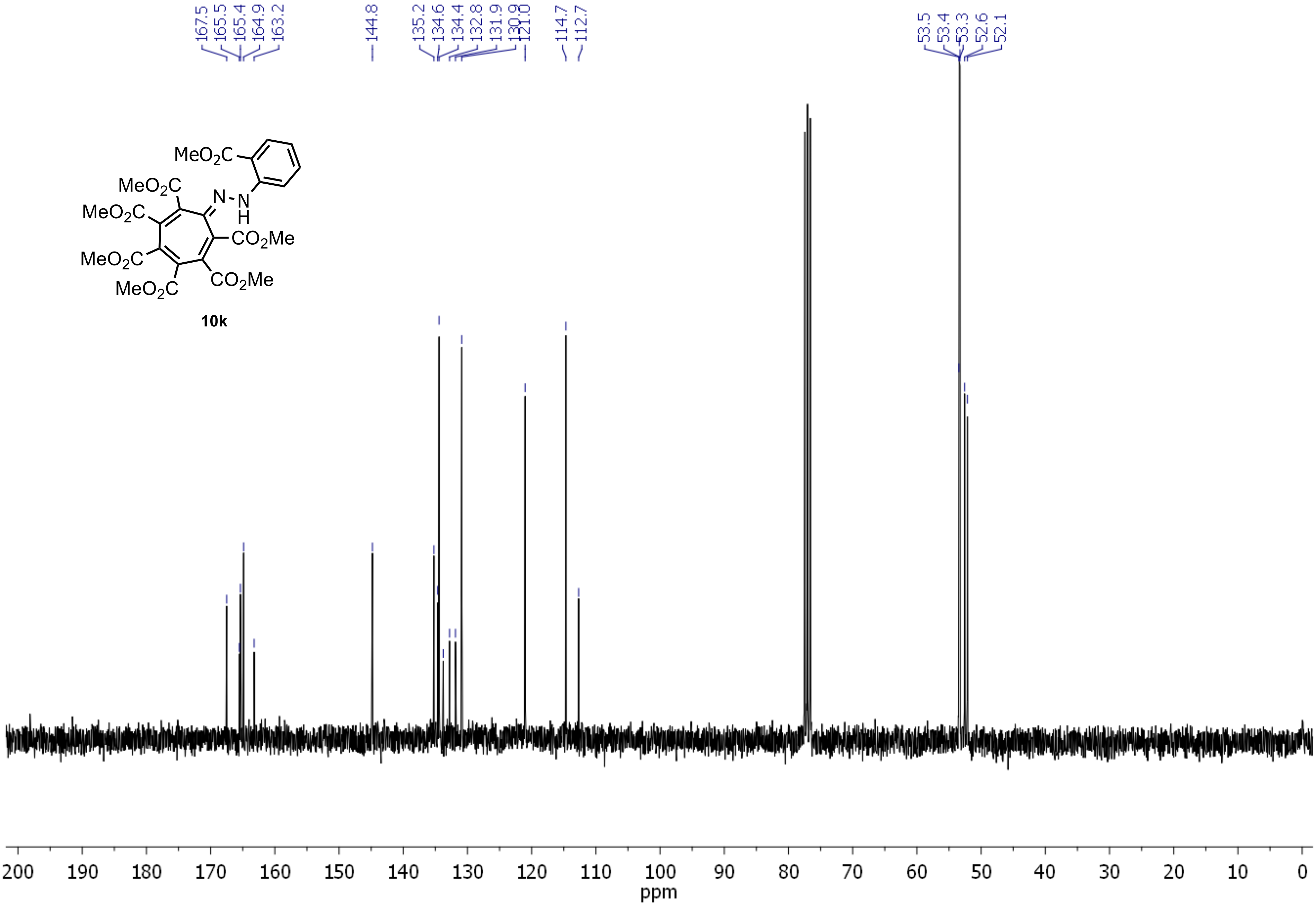
167.5
165.5
165.4
164.9
163.2
144.8
135.2
134.6
134.4
132.8
131.9
130.9
121.0
114.7
112.7
53.5
53.4
53.3
52.6
52.1
MeO2C
MeO2C
MeO2C
N
N
H
CO2Me
MeO2C
MeO2C
CO2Me
10k
200 190 180 170 160 150 140 130 120 110 100 90 80 70 60 50 40 30 20 10 0
ppm

6. References

(1) Ilyushchenko, M. K.; Salikov, R. F.; Sokolova, A. D.; Litvinenko, V. V.; Belyy, A. Yu.; Platonov, D. N.; Tomilov, Y. V. *J. Org. Chem.* **2023**, *88*, 5661–5670. doi:10.1021/acs.joc.3c00142

(2) Gund, S. H.; Balsane, K. E.; Nagarkar, J. M. *Tetrahedron Lett.* **2017**, *58*, 2936–2939. doi:10.1016/j.tetlet.2017.06.045

(3) Heinrich, M. R.; Blank, O.; Ullrich, D.; Kirschstein, M. *J. Org. Chem.* **2007**, *72*, 9609–9616. doi:10.1021/jo701717k

(4) Neese, F. *WIREs Comput. Mol. Sci.* **2012**, *2*, 73–78. doi:10.1002/wcms.81

(5) Pracht, P.; Grimme, S.; Bannwarth, C.; Bohle, F.; Ehlert, S.; Feldmann, G.; Gorges, J.; Müller, M.; Neudecker, T.; Plett, C.; Spicher, S.; Steinbach, P.; Wesołowski, P. A.; Zeller, F. *J. Chem. Phys.* **2024**, *160*, 114110. doi:10.1063/5.0197592

(6) Santra, G.; Sylvetsky, N.; Martin, J. M. L. *J. Phys. Chem. A* **2019**, *123*, 5129–5143. doi:10.1021/acs.jpca.9b03157

(7) Lu, T.; Chen, F. *J. Comput. Chem.* **2012**, *33*, 580–592. doi:10.1002/jcc.22885

(8) Knizia, G.; Klein, J. E. M. N. *Angew. Chem. Int. Ed.* **2015**, *54*, 5518–5522. doi:10.1002/anie.201410637

(9) Ramos-Cordoba, E.; Postils, V.; Salvador, P. *J. Chem. Theory Comput.* **2015**, *11*, 1501–1508. doi:10.1021/ct501088v
